# Suppression of Methanol and Formate Crossover through Sulfanilic‐Functionalized Holey Graphene as Proton Exchange Membranes

**DOI:** 10.1002/advs.202304082

**Published:** 2023-09-08

**Authors:** Samuel Jeong, Tatsuhiko Ohto, Tomohiko Nishiuchi, Yuki Nagata, Jun‐ichi Fujita, Yoshikazu Ito

**Affiliations:** ^1^ Institute of Applied Physics Graduate School of Pure and Applied Sciences University of Tsukuba 1‐1‐1 Tennodai Tsukuba Ibaraki 305‐8571 Japan; ^2^ Department of Materials Design Innovation Engineering Nagoya University Furo‐cho Chikusa‐ku Aichi 464‐8603 Japan; ^3^ Graduate School of Engineering Science Osaka University 1‐3 Machikaneyama Toyonaka Osaka 560‐8531 Japan; ^4^ Department of Chemistry Graduate School of Science Osaka University 1‐1 Machikaneyama Toyonaka Osaka 560‐0043 Japan; ^5^ Max Planck Institute for Polymer Research Ackermannweg 10 55128 Mainz Germany

**Keywords:** crossover, formic acid, functional group introduction, methanol, permeability, proton exchange membranes

## Abstract

Proton exchange membranes with high proton conductivity and low crossover of fuel molecules are required to realize advanced fuel‐cell technology. The selective transportation of protons, which occurs by blocking the transportation of fuel molecules across a proton exchange membrane, is crucial to suppress crossover while maintaining a high proton conductivity. In this study, a simple yet powerful method is proposed for optimizing the crossover‐conductivity relationship by pasting sulfanilic‐functionalized holey graphenes onto a Nafion membrane. The results show that the sulfanilic‐functionalized holey graphenes supported by the membrane suppresses the crossover by 89% in methanol and 80% in formate compared with that in the self‐assembled Nafion membrane; an ≈60% reduction is observed in the proton conductivity. This method exhibits the potential for application in advanced fuel cells that use methanol and formic acid as chemical fuels to achieve high energy efficiency.

## Graphical abstract

Proton exchange membranes with high proton conductivity and low crossover of fuel molecules are required to realize advanced fuel‐cell technology. The sulfanilic‐functionalized holey graphene, which is supported by the membrane suppresses the crossover by 89% in methanol and 80% in formate compared with that in the self‐assembled Nafion membrane; an ≈60% reduction is observed in the proton conductivity.

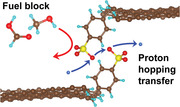

## 1 Introduction

Recycling carbon dioxide (CO_2_) and utilizing fuel cells are crucial to achieve carbon neutrality for a sustainable society.^[^ ^1^ ^]^ A promising technology to reduce CO_2_ emissions is the electrochemical CO_2_ reduction reaction that uses renewable energy to produce energy carriers, including methanol and formic acid. These energy carriers can be directly used as chemical fuels for fuel cells, without requiring dehydrogenation processes.^[^ ^2^, ^3^ ^]^ These fuel cells are called direct methanol fuel cells (DMFC)^[^ ^4^, ^5^ ^]^ and direct formic acid fuel cells (DFAFC).^[^ ^6^ ^−^ ^8^ ^]^ A high proton conductivity and low crossover of fuel molecules are required in these fuel cells. Nafion is the standard membrane used in fuel cells because it exhibits excellent proton conductivity (proton conductivity: 10−100 mS cm^−1^ and areal proton conductivity: 0.2−3.3 S cm^−2^).^[^ ^8^ ^−^ ^10^ ^]^ However, the Nafion membrane displays a large crossover from the anode to cathode chambers (methanol: 1.0−5.0 × 10^−6^ cm^2^ s^−1^; formic acid: 0.68 × 10^−6^ cm^2^ s^−1^) that prevents the practical use of DMFC and DFAFC.^[^ ^11^ ^]^ The crossover of fuel molecules in a full‐cell system is caused by electro‐osmosis and pressure/concentration gradients between the cathode and anode chambers.^[^ ^12^, ^13^ ^]^

These causative factors decrease the efficiency of the electrochemical CO_2_ reduction reaction, trigger fuel loss, and generate a mixed potential due to fuel oxidation at the cathode in the DMFC and DFAFC.^[^ ^14^ ^−^ ^16^ ^]^ A simple way to suppress the crossover is to increase the membrane thickness; however, this approach has two drawbacks. First, increasing the membrane thickness inevitably increases the size of the fuel‐cell units and associated material costs. Second, the internal resistance to proton transport through the membrane increases with increasing membrane thickness, which decreases energy efficiency.^[^ ^7^, ^16^, ^17^ ^]^

The former may be mitigated by improving the selectivity of the membranes to molecules and ions in the cell,^[^ ^12^, ^18^ ^]^ by installing metal–organic frameworks (MOFs),^[^ ^19^ ^]^ graphene,^[^ ^2^, ^20^ ^−^ ^24^ ^]^ and other 2D nanomaterials^[^ ^10^, ^12^ ^]^ on the surface of the Nafion membrane. In particular, graphene sheets have been reported to block large molecules from penetrating the graphene lattice while allowing protons to penetrate.^[^ ^10^, ^21^, ^25^, ^26^ ^]^ The combination of Nafion and graphene successfully suppressed the crossover of fuel molecules such as methanol and formic acid.^[^ ^2^, ^22^, ^23^, ^26^ ^−^ ^28^ ^]^ However, such membranes failed to meet the minimum requirement of proton conductivity in the fuel cells (i.e., 10% proton conductivity, ≈0.1 S cm^−1^ or 1000 mS cm^−2^, of Nafion membranes).^[^ ^29^ ^]^ To date, attempts to maintain a high proton conductivity have been unsuccessful, and advanced fuel cells have not been used practically. A balance is required between the crossover of fuel molecules and proton conductivity.

In this study, we achieved an excellent balance between the crossover of fuel molecules and proton conductivity by fabricating holey graphene with physical and chemical modifications. The incorporation of 5−20 nm holes on graphene markedly increased the proton conductivity, whereas the introduced functional groups on the graphene suppressed the crossover of fuel molecules. The membrane fabricated with the sulfanilic functional group suppressed the crossover by >80% with a slightly reduced proton conductivity compared with the Nafion membranes. It is acknowledged that sulfanilic groups suppress the crossover but increase the proton conductivity.^[^ ^2^, ^22^ ^]^ Moreover, density functional theory (DFT) calculations indicated that graphene comprising sulfanilic functional groups increased the energy barrier of the transport of methanol and formic acid and provided a hopping conduction pathway for protons through the membranes. This graphene‐based technology is a simple yet powerful method for the rational design of fuel membranes.

## 2 Results

### 2.1 Membrane Characterization

A high‐quality single graphene layer (1GL) and a single holey graphene layer (1hGL) were synthesized on a Cu sheet with the chemical vapor deposition (CVD) method with/without Si nanoparticles (≈20 nm in diameter) on the Cu sheet before the CVD process (Figures S1–S3, Supporting Information).^[^ ^30^ ^]^ For the single‐functional graphene layer (1fGL), we chemically functionalized 1hGL with sulfanilic acids to introduce benzenesulfonic acid groups (─C_6_H_4_─SO_3_H) on the edges of the holes (see the Experimental Section for details).

**Figure 1:**
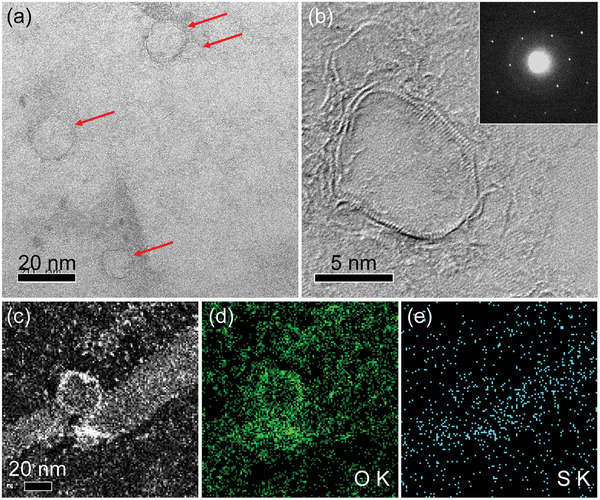
Morphological characterizations of sulfanilic‐functionalized holey graphene. a) Transmission electron microscopy and b) high‐resolution transmission electron microscopy images of hole regions on 1fGL. The inset of (b) shows electron diffraction patterns. Scanning transmission electron microscopy images of 3fGL for c) dark field‐scanning transmission electron microscopy, energy dispersive X‐ray spectroscopy (EDS) mapping of d) O, and e) S.

The transmission electron microscopy (TEM) images indicate that the holes on 1hGL and 1fGL are 5–20 nm in diameter (**Figure** **1**a; Figures S2 and S4, Supporting Information). The high‐resolution TEM (HRTEM) images of the 1fGL show that a 0.5 nm width fringe is present around the hole; by contrast, the other graphene regions are highly crystalline (Figure 1b). The corresponding electron diffraction patterns show sharp diffraction spots, indicating that 1hGL and 1fGL are highly crystalline (Figure 1b; Figure S2, Supporting Information). The dark‐field scanning TEM (DF‐STEM) images indicate that the area ratio of the hole and non‐hole area are ≈12.0 ± 1.5% on average (Figure S4, Supporting Information). The elemental distributions of the S and O atoms in 1fGL were confirmed using in situ energy dispersive X‐ray spectroscopy (EDS). The concentrations of S and O in the benzenesulfonic acid groups are qualitatively twice as high in the near‐hole regions compared with those in the plane regions (Figure 1c–e).

**Figure 2:**
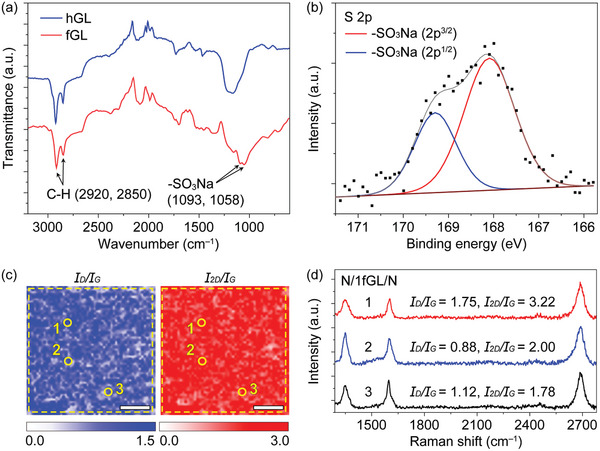
Structural characterizations of sulfanilic‐functionalized holey graphene. a) Fourier transform infrared spectra of 1hGL and 1fGL. b) XPS S 2p spectra for the 1fGL. c) Raman map images of N/1fGL/N on a window‐attached Si_3_N_4_ chip. The dotted square indicates the window area. d) Raman spectra of N/1fGL/N were collected at the 1, 2, and 3 positions in (c). Scale bar: 5 µm.

The presence of benzenesulfonic acid groups in 1fGL was further confirmed using Fourier transform infrared spectroscopy (FT‐IR) and X‐ray photoelectron spectroscopy (XPS). The FT‐IR spectra of the fGL samples show fingerprints of the sulfanilic‐functionalized group at 1058 and 1093 cm^−1^ (─SO_3_Na),^[^ ^22^, ^31^ ^]^ which are not observed in the spectra of the hGL samples (**Figure** **2**a). Note that ─SO_3_H groups are observed as ─SO_3_Na groups in the dried state. The XPS S 2p spectra of the 1fGL confirm the chemical binding state of the sulfanilic‐functionalized group on graphene (169.2 and 168.0 eV).^[^ ^32^ ^]^ The atomic concentration of the S atoms is ≈2.49 at.% on the 1fGL samples (Figure 2b; Figure S5, Supporting Information).

The spatial Raman mapping images of a graphene layer (1GL, 1hGL, 1fGL) sandwiched by two spin‐coated Nafion membranes on a window‐attached Si_3_N_4_ chip (window size: 20 × 20 µm) were examined to study the relationship between the functionalization and level of defects. The Nafion/graphene/Nafion‐sandwiched membranes have ≈2 µm thickness with 80 nm maximum roughness and 4% roughness ratio (Figures S6–S8, Supporting Information) (see the Experimental Section for more detail). We abbreviated the Nafion sandwiched mono‐layer graphene membranes as N/1GL/N, N/1hGL/N, and N/1fGL/N. The mapping image of the N/1fGL/N shows 87% of mono‐layer graphene characteristics (high 2D‐band to G‐band (*I*_2D_/*I*_G_) intensity ratio: 1.78−3.22). Moreover, the N/1fGL/N show a relatively high defect level (D‐band to G‐band (*I*_D_/*I*_G_) intensity ratio: 0.88−1.75) (Figure 2c,d) compared with that of N/1GL/N (*I*_2D_/*I*_G_:3.64−4.00, *I*_D_/*I*_G_:0.03−0.07) and N/1hGL/N (*I*_2D_/*I*_G_:1.98−3.63, *I*_D_/*I*_G_:0.46−0.91) (Figure S3, Supporting Information). The high defect level is induced by the functionalized groups. Furthermore, bi‐layer (namely, N/2GL/N, N/2hGL/N, and N/2fGL/N) and tri‐layer (namely, N/3GL/N, N/3hGL/N, and N/3fGL/N) graphene sandwiched by two spin‐coated Nafion sheets were prepared by manually stacking mono‐layer graphene (1GL, 1hGL, and 1fGL) (Figure S6, Supporting Information) to elucidate the mechanisms prevailing in the different configurations (Figures S9 and S10, Supporting Information).

### 2.2 Design Principles of Fabricated Membranes

**Figure 3:**
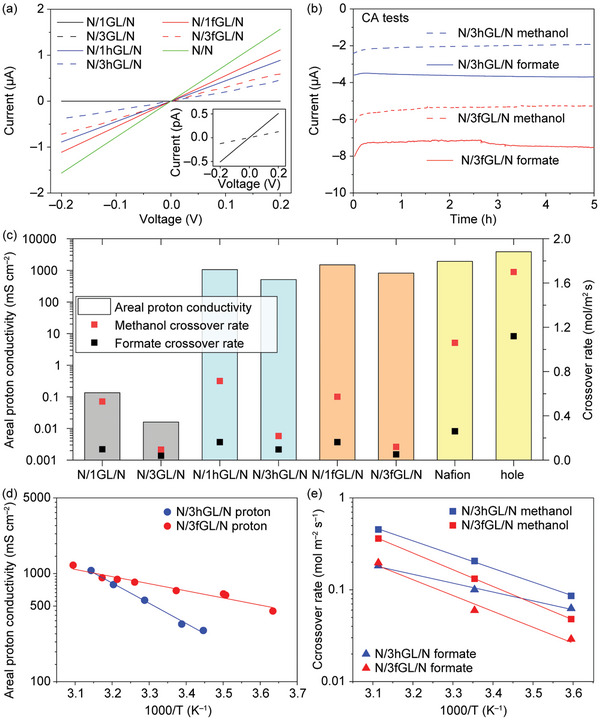
Electrochemical properties of graphene membranes. a) Current–voltage characteristics of the proton current through various graphene membranes. b) Proton current collected by chronoamperometry (CA) measurements at a cell voltage of −1.6 V. c) Summary of areal proton conductivities and crossover rates of methanol and formate through various graphene membranes. Temperature dependence Arrhenius‐type plots of d) areal proton conductivity at −1.0 V, and e) methanol and formate crossover rates for N/3hGL/N and N/3fGL/N at −1.6 V.

We investigated the penetration of protons and fuel molecules (methanol and formic acid) through various graphene membranes (Figures S11–S16, Supporting Information). For the electrochemical measurements, the N/xGL/N, N/xhGL/, and N/xfGL/N (x refers to the number of graphene layer) placed on the open‐windowed Si_3_N_4_ chip to serve both as proton exchange and separating membrane in an H‐type cell.^[^ ^25^ ^]^ The Si_3_N_4_ chip was reinforced with a polyethylene terephthalate (PET) sheet to fix it in the H‐type cell to physically isolate the working and counter electrode chambers of the H‐type cell (Figure S11, Supporting Information). To investigate proton penetration, we immersed the graphene‐suspended membrane device in a sulfuric acid solution (0.05 m) and performed electrochemical tests using a two‐electrode method with Pt mesh electrodes. **Figure** **3**a shows an example of the current–voltage (*I*–*V*) characteristics measured in the devices using the N/xGL/N, N/xhGL/N, and N/xfGL/N. The measured proton current, *I*, varies linearly with the bias voltage, *V*. The conductance *S* ( = *I*/*V*) divided by the effective membrane area (*A*) is discussed below as the areal proton conductivity σ ( = *S*/*A*).^[^ ^10^, ^25^, ^33^ ^]^ The crossover rate of the fuel molecules was calculated from the quantity of fuel molecules that crossed over from the working electrode chamber containing methanol or formate (formic acid exists as formate in acid) to the counter electrode chamber using chronoamperometry (CA) (Figure 3b,c; Figures S14–S16, Supporting Information).

To prevent crossover of the fuel molecules, we introduced 1GL by sandwiching the graphene sheet using a Nafion membrane (i.e., N/1GL/N) (Figure S6, Supporting Information). The crossover rates of methanol and formate are 0.530 and 0.098 mol m^−2^s^−1^, respectively, indicating that the combination of 1GL and Nafion membrane suppresses the crossover markedly compared with our Nafion/Nafion membrane (namely, N/N), which was prepared from Nafion solution (Figure 3c). However, the areal proton conductivity is largely suppressed in the N/1GL/N (0.135 mS cm^−2^) compared with that in the N/N (1930 mS cm^−2^) (Figure 3c; Table S1, Supporting Information). Our data are consistent with other data obtained for the reported Nafion membrane (200–3300 mS cm^−2^).^[^ ^4^, ^10^, ^25^ ^]^

To improve the areal proton conductivity, we created holes with radii of 5–20 nm on 1GL (Figure S1, Supporting Information). As expected, the areal proton conductivity of N/1hGL/N improves markedly from 0.135 to 1060 mS cm^−1^, which is comparable with that of the N/N. Surprisingly, the crossover rates of methanol and formate are 0.715 and 0.163 mol m^−2^s^−1^, respectively, and are comparable with those of N/1GL/N; they are markedly improved compared with those of the N/N (methanol: 1.060 mol m^−2^s^−1^ and formate: 0.261 mol m^−2^s^−1^). These results indicate that the holes on the graphene sheet enable the selective transport of the molecules and protons.

To further optimize the performance of the membrane for fuel cells, we functionalized holey graphene with sulfanilic groups. Figure 3c summarizes the areal proton conductivities and crossover rates of methanol and formate through the various graphene membranes. The areal proton conductivity of N/1fGL/N is 1500 mS cm^−2^, which is higher than that of N/1hGL/N (1060 mS cm^−1^); the crossover for methanol and formate is 0.573 and 0.163 mol m^−2^s^−1^, respectively; this result is comparable with that obtained for N/1hGL/N (methanol: 0.715 mol m^−2^s^−1^ and formate: 0.163 mol m^−2^s^−1^). Therefore, the sulfanilic functional groups on 1hGL (i.e., 1fGL) maintain 77.7% proton conductivity and suppress the crossover rates for methanol and formate by 45.9% and 37.5%, respectively, compared with those of the N/N (Tables S1 and S2, Supporting Information).

**Figure 4:**
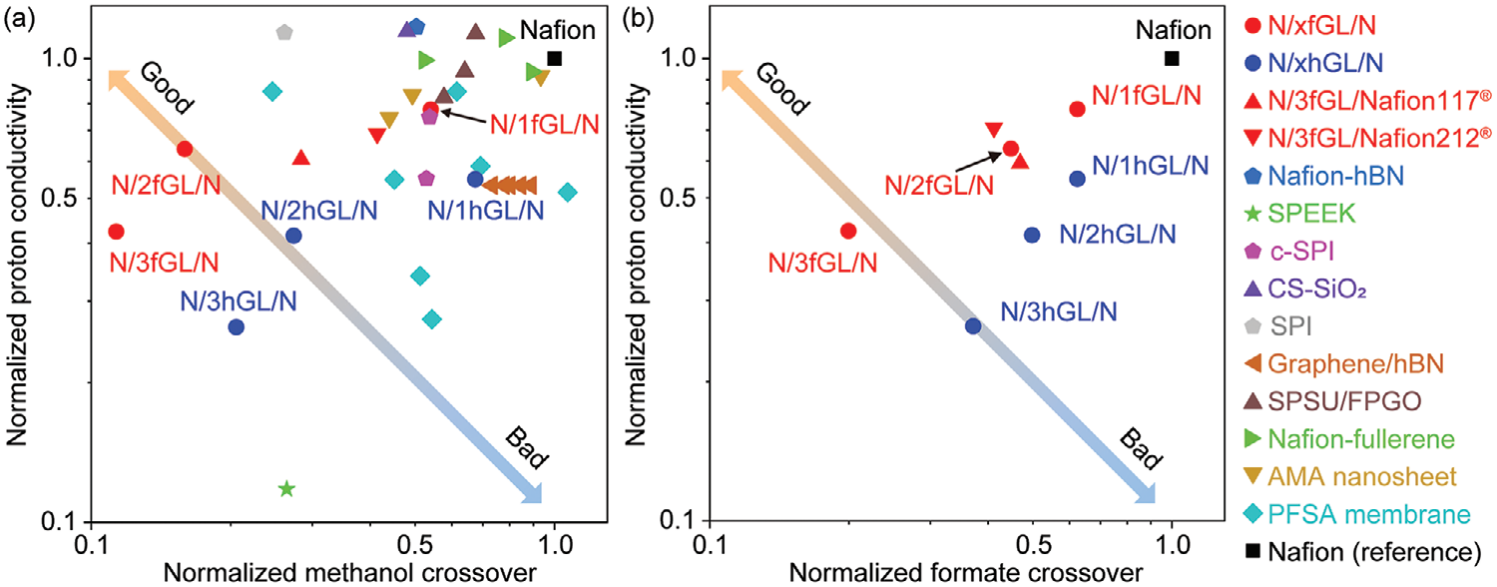
Summary of the membrane performance. a) Normalized proton conductivity and methanol crossover rate with Graphene/hBN,^[^ ^12^ ^]^ Amino‐MIL‐53(Al) (AMA) nanosheet,^[^ ^15^ ^]^ composite of functional polysiloxane brushes modified graphene oxide and sulfonated polysulfone (SPSU/FPGO),^[^ ^28^ ^]^ perfluorosulfonic acid (PFSA) membrane,^[^ ^34^ ^]^ Nafion‐fullerene,^[^ ^35^ ^]^ Nafion‐hBN,^[^ ^36^ ^]^ sulfonated polyether ether ketone (SPEEK),^[^ ^37^ ^]^ sulfonated polyimide(c‐SPI),^[^ ^38^ ^]^ sulfonated SiO_2_ nanoparticles composite (CS‐SiO_2_),^[^ ^39^ ^]^ and sulfonated polyimide (SPI).^[^ ^40^ ^]^ b) Normalized proton conductivity and formate crossover rate. The proton conductivities and methanol/formate crossover rates of each sample were normalized by the proton conductivity and methanol/formate crossover rate of Nafion membrane measured in each report. The “x” in the N/xfGL/N and N/xhGL/N refers to the number of graphene layers.

After recognizing that the N/1fGL/N showed a marked improvement in the membrane performance compared with the performance of N/1GL/N and N/1hGL/N, we optimized the number of graphene layers using the same experimental conditions. Among the tri‐layer graphenes, N/3fGL/N displays the highest areal proton conductivity compared with those of N/3GL/N (0.016 mS cm^−2^), N/3hGL/N (509 mS cm^−2^), and N/3fGL/N (818 mS cm^−2^); the areal proton conductivity is approximately half that of the N/N (Table S1, Supporting Information). N/3fGL/N exhibits lower methanol and formate crossover rates (0.120 and 0.052 mol m^−2^s^−1^, respectively) than those of N/3hGL/N; the crossover rate of N/3fGL/N is comparable with that of N/3GL/N (Figure 3c; Tables S1 and S2, Supporting Information). Notably, N/3fGL/N maintains 42.3% of the proton conductivity of the N/N. N/3fGL/N suppresses the methanol and formate crossover rates by 88.7% and 80.0%, respectively, compared with that of the N/N; N/3fGL/N exhibits an extremely low crossover of fuel molecules and almost meets the required target proton conductivity (≈1000 mS cm^−2^)^[^ ^10^ ^]^ for fuel cell operations. Notably, N/3fGL/N exhibits an areal proton conductivity of 1196 mS cm^−2^ at 50 °C (i.e., practical operation temperature), which completely meets the requirement of hydrogen‐type fuel cells (Figure 3d,e).^[^ ^10^ ^]^ N/3fGL/N (or N/2fGL/N) demonstrates the best balance between the proton conductivity and suppression of the fuel crossover (**Figure** **4**; Tables S3 and S4, Supporting Information) among the reported methanol crossover suppression membranes.^[^ ^12^, ^15^, ^34^ ^−^ ^40^ ^]^

Subsequently, we investigated the membrane stability by scanning electron microscope (SEM) and Raman spectroscopy. The cross‐sectional SEM images after the crossover tests confirmed that the membranes maintained their dimensions (Figure S17, Supporting Information) and the flat surface structures after the crossover tests (Figure S18, Supporting Information). The Raman mapping after the test indicated that the graphene characters were preserved for the entire membrane area (Figure S19, Supporting Information). These results suggest that graphene layers sandwiched with Nafion membranes are highly stable during the tests.

To establish the impact of the thickness of the Nafion membrane and suppression of crossover by 3fGL, we investigated areal proton conductivity and methanol/formate crossover rates using commercially available Nafion 212 (thickness; 50 µm) and Nafion 117 (thickness; 180 µm) compared with those of our Nafion (N/N, thickness: 2 µm). Notable reductions of the crossover rate (Nafion 212: 0.0425 mol m^−2^ s^−1^ for methanol and 0.0146 mol m^−2^ s^−1^ for formate; Nafion 117: 0.0184 mol m^−2^ s^−1^ for methanol and 0.0015 mol m^−2^s^−1^) and proton conductivity (117.2 mS cm^−2^ for Nafion 212, 88.7 mS cm^−2^ for Nafion 117 in our experimental setting) are observed with an increase of the thickness of the membrane from 2 to 180 µm (Figure S20 and Tables S1 and S2, Supporting Information). The crossover suppression ratios of Nafion 117 to N/N are 98.3% in methanol and 99.4% in formate and the areal proton conductivity reduction ratio of Nafion 117 to N/N is 95.4%, which are 35% higher than those of the Nafion membranes (N/3fGL/N to N/N). Thus, we confirmed that the increase of membrane thickness markedly reduces the areal proton conductivity while preventing crossover. Next, we examined the performances of N/3fGL‐pasted Nafion 212 and Nafion 117 membranes (namely, N/3fGL/Nafion 212 and N/3fGL/Nafion 117). The crossover suppression ratios of N/3fGL/Nafion 212 or 117 to Nafion 212 or 117 are 58.5% in methanol and 58.7% in formate for N/3fGL/Nafion 212 and 71.5% in methanol and 53.2% in formate for N/3fGL/Nafion 117 with 39.2% and 31.2% reduction of the areal proton conductivity, respectively (Tables S1 and S2, Supporting Information). These results indicated that our crossover suppression technology is applicable to commercially available membranes.

### 2.3 Mechanism of the High Selectivity of 3fGL

We experimentally examined the energy barriers for proton penetration as well as methanol and formate penetrations through N/3fGL/N and N/3hGL/N using Arrhenius‐type plots in the temperature range of 5 to 50 °C (Figure 3d,e; Figure S21 and Table S5, Supporting Information).^[^ ^10^ ^]^ The apparent energy barriers of proton penetration at the −1.0 V bias voltage estimated from the Arrhenius‐type plots are 0.37 and 0.13 eV for N/3hGL/N and N/3fGL/N, respectively. These results imply that the sulfanilic functional groups reduce the energy barrier for proton penetration. The Arrhenius‐type plots of the crossover rates of methanol and formate obtained from 5 h CA measurements are displayed in Figure 3d. The apparent energy barriers of methanol crossover at the −1.6 V bias voltage are 0.30 and 0.36 eV for N/3hGL/N and N/3fGL/N, respectively (Figure 3e). The apparent barrier energies of the formate crossover at the −1.6 V bias voltage are 0.19 and 0.34 eV for N/3hGL/N and N/3fGL/N, respectively (Figure 3e). The apparent energy barrier increases for the crossover of both methanol and formate upon the introduction of the sulfanilic functional groups in the hole regions of the graphene.

**Figure 5:**
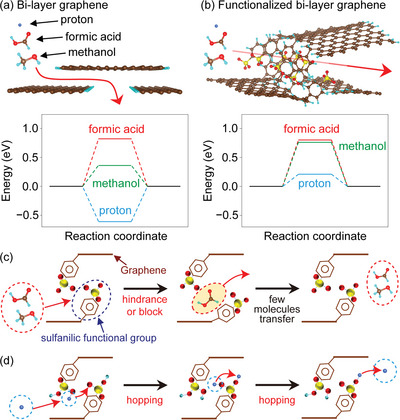
Computational simulations of crossover of molecules. Interlayer models and energy diagram for proton penetration, methanol, and formic acid penetration through a) bi‐layer graphene and b) sulfanilic‐functionalized bi‐layer graphene. Schematic illustrations of c) fuel molecule and d) proton penetration through the (b) model.

We performed DFT calculations to examine the effect of the sulfanilic functional groups (attached to the graphene holes) on the molecular sieving effect. First, we investigated hole size dependence of the proton and fuel molecular penetration energy barriers and confirmed that the energy barriers on a graphene model with a hole ≥1 nm diameter are negligible (Figure S22; Table S6, Supporting Information). To this end, we computed the energy barrier for the process, wherein a proton/methanol/formic acid molecule passes through a nanoconfined space created by the graphene layers (**Figure** **5**a,b; Figure S22–S26 and Tables S6–S8, Supporting Information). The energy barriers for the holey graphene comprising sulfanilic functional groups are 0.21, 0.76, and 0.80 eV for proton, methanol, and formic acid penetrations, respectively; the energy barriers for the holey graphene excluding sulfanilic groups are −0.63 eV, 0.36, and 0.82 eV for proton, methanol, and formic acid penetrations, respectively (Figure 5a–d). Importantly, the positive energy barriers of the methanol and formate models indicate that external energy is required to pass through the graphene layers. However, the negative energy barrier of the protons in the graphene sheets that exclude the sulfanilic groups indicates that protons are adsorbed on the graphene edges. In the presence of sulfanilic functional groups, the protons are easily transferred between the sulfanilic functional groups via the Grotthuss mechanism (Figure 5d).^[^ ^41^ ^]^ These results confirm that the crossover is suppressed, and proton conductivity is maintained on the sulfanilic‐functionalized graphene membranes.

## 3 Discussion

We investigated the effect of sulfanilic‐functionalized holey graphene on areal proton conductivity and methanol and formic acid crossover rates by combining electrochemical measurements and DFT‐calculated energy barriers. The fGL membranes notably suppressed crossover while maintaining high areal proton conductivity. The improvements could be attributed to the following factors: i) introduction of holes into the graphene lattice (i.e., hGL) provides multiple proton penetration pathways between graphenes; ii) sulfanilic‐functionalized groups on holey graphene (i.e., fGL) enable the formation of new proton conduction pathways by the transfer of H^+^ ions via the Grotthuss mechanism and accelerate the proton hopping conduction on the sulfanilic functional groups in interplanar graphenes (indeed, hGL without sulfanilic functional groups displayed high crossover rates, implying that the protons in the form of H_3_O^+^ are accompanied by water/fuel molecules during the proton penetration, i.e., water/fuel electroosmotic drag effect); iii) apparent energy barrier of the crossover of both methanol and formate increases upon the introduction of the bulky net‐like sulfanilic functional groups on the molecular migration pathway; iv) negatively charged sulfanilic functional groups introduce a steric hindrance and block anionic molecules, thereby inhibiting the migration of methanol and formate, although allowing the migration of small and positively charged protons. The synergistic effects of introducing holes and sulfanilic‐functionalized groups into graphene play a crucial role in balancing selective proton transfer and suppressing the crossover of fuel molecules.

## 4 Conclusion

In this study, we systematically revealed the penetration mechanisms of protons, methanol, and formate molecules through sulfanilic‐functionalized holey graphene. The results indicated that proton conductivity and crossover of fuel molecules could be tuned by introducing nanometer‐sized holes in graphene, functionalizing holey graphene with sulfanilic functional groups, and adjusting the number of graphene layers. The crossover suppression and maintenance of high areal proton conductivity were realized, and all the membranes reported previously could be improved by simply pasting the graphene membrane on their surface, thereby reducing the material cost of Nafion membrane and the cell volume. Our findings should contribute to the development of electro synthetic cells for electrochemical CO_2_ reduction and advanced fuel cells such as DMFCs and DFAFCs, and can be used for other chemical fuels such as ammonia, urea, and hydrogen peroxide by changing proper functional groups in advanced fuel cells for facilitating a carbon‐neutral society.

## 5 Experimental Section

### Synthesis of Graphene, Holey Graphene, and Functionalized Holey Graphene

1GL was grown on a Cu foil (99.8%, 25 µm thickness, Alfa Aesar, UK) with a conventional CVD method. The Cu foil was inserted into the center of a quartz tube (φ30 × φ27 × 1000 mm) in a furnace. The tube was heated at 1000 °C for 1 h under an atmosphere of H_2_ (100 sccm, 99.99%) and Ar (200 sccm, 99.995%). Thereafter, mono‐layer graphene was grown with an additional flow of CH_4_ (20 sccm, 99.995%) for 30 min. After the graphene growth, the furnace was immediately opened, and the quartz tube was cooled down to room temperature (25 °C) with a fan. 1hGL was synthesized by a similar method as that used for 1GL, but SiO_2_ nanoparticles (18–27 nm in diameter, ST‐50, 48 wt.%, Nissan Chemical Industries, Japan) were distributed on the Cu foil before the CVD process. A purchased SiO_2_ solution was diluted to 1×10^−4^ wt.% with ultrapure water and then it was further diluted with 2‐propanol (water:2‐propanol=1:1 (v/v)) to control the density of holes. Thereafter, 10 µL of the dispersion was added dropwise onto a 1 cm^2^ copper foil and dried. Subsequently, 1hGL was obtained under the same CVD conditions as those used for 1GL. For 1fGL, the synthesized 1hGL on the Cu foil was immersed in a mixed solution of 50 mL of sulfanilic acid solution (0.1 m) and 10 mL of NaNO_2_ solution (0.01 m), and heated at 70 °C for 12 h. After heating, the blackened solution was replaced with pure water several times for washing and the 1fGL on the Cu foil was dried. The as‐synthesized graphene on the Cu foil was chemically etched away in 50 mL of Fe(NO_3_)_3_ (0.25 m) solution and the isolated graphene was manually stacked to prepare graphene membranes (please refer to the Supporting Information for more detail and Figures S1‐S6, Supporting Information).

### Characterization of Graphene Samples

The morphology and microstructure of the as‐synthesized graphene were characterized using a SEM (JEOL JCM‐7000) and TEM (JEOL JEM‐2100F and JEM‐ARM200F‐B) with 80 kV accelerating voltage to prevent damage to the graphene, and equipped EDS (SDD Type, Detection surface area 30 mm^2^, Solid angle 0.26 sr). Raman spectra were obtained using a Renishaw InVia Reflex with an incident wavelength of 532.5 nm. Raman measurements were performed using this Si_3_N_4_ chip‐supported Nafion/graphenes/Nafion membrane. The obtained Raman spectra were normalized by the maximum peak intensity. Fourier transform infrared (FT‐IR) spectroscopy (ThermoFisher (Nicolet iS 50) FT‐IR spectrometer) with the wavenumber ranging from 3200 to 600 cm^−1^ was performed using an attenuated total reflection method. To obtain a clear signal intensity from the FT‐IR, the study measured 30 layers stacked 1hGL and 1fGL samples, respectively. Surface chemical states were studied using XPS (AXIS ultra DLD, Shimadzu) with an Al Ka and X‐ray monochromator. All samples were transferred on a Si_3_N_4_ chip or Cu TEM mesh grid for the measurements. The surface roughness measurements were performed on a thermal oxide Si wafer substrate (< 1 nm roughness, Canosis Co. Ltd, Japan) by AFM (Hitachi, AFM5000II).

### Areal Proton Conductivity Measurements

An electrochemical workstation (Biologic, VSP‐300) equipped with an H‐type cell was used for electrochemical measurements. The schematic of the H‐type cell configurations for electrochemical measurements of proton penetration was shown in Figure S11 (Supporting Information). A PET sheet separator with a window‐attached Si_3_N_4_ chip (window size: 20 × 20 µm) fixed in the center was used to isolate the working electrode and counter electrode chambers filled with H_2_SO_4_ (0.05 m) solution. Proton conductivity measurements were performed in a two‐electrode system, where both Pt meshes (35 × 25 mm) were used as a working and counter electrode. Note that the proton conductivity was checked using aqueous H_2_SO_4_ (0.05 m) and aqueous H_2_SO_4_ (0.5 m) solution and there were no obvious differences (Figure S12, Supporting Information). Areal proton conductivity was investigated by measuring the current–voltage (*I*–*V*) characteristics with a bias voltage of +200 to −200 mV, applied between two Pt mesh electrodes. The temperature dependence of the areal proton conductivity was measured from −1.0 to +0.2 V after a 5 min waiting time to stabilize the measurement temperature.

### Crossover Measurements for Methanol and Formate

A 30% volumetric concentration of methanol or formic acid in the aqueous H_2_SO_4_ (0.05 m) solution were used as the electrolytes for the working electrode for chronoamperometry (CA) measurements at −1.6 V using a two‐electrode system. After the 5 h CA test, the electrolyte in the counter electrode chamber was collected, and a quantitative analysis of methanol and formate was performed using nuclear magnetic resonance (NMR) and gas chromatography (GC). The crossover rate from the working electrode to the counter electrode chamber was calculated using the following Equation:

(1) $$\mathrm{Crossover}\;\mathrm{rate}\;\mathrm{of}\;\mathrm{fuel}\;\mathrm{molecular}\;\;\left[\frac{\mathrm{mol}}{m^{2}s}\right]=\frac{c\times V}{A\times t}$$

where *c* is the detected molar concentration of methanol or formate (mol/mL), *V* is the electrolyte volume (mL), *A* is the membrane area in the chip window (m^2^), and *t* is the measurement time (s).

### Density Functional Theory Calculation

DFT calculations were performed via the CP2K program.^[^ ^42^ ^]^ The Becke‐Lee‐Yang‐Parr (BLYP)^[^ ^43^ ^]^ exchange‐correlation functional was used. Double‐zeta valence plus polarization (DZVP) basis sets were used. The core electrons were described by the Goedecker‐Teter‐Hutter pseudopotential.^[^ ^44^ ^]^ The real‐space density cut‐off was set to 320 Ry. The van der Waals correction was included via Grimme's D3 method.^[^ ^45^ ^]^ The simulation cell lengths in the *x*‐, *y*‐, and *z*‐directions were 25.56, 24.595, and 50 Å.

The graphene interlayer model was constructed using two overlapping graphene nanoribbons having a width of ≈15 Å. The edge of the graphene nanoribbon was terminated with hydrogen atoms. The sulfanilic‐functionalized graphene interlayer was created by replacing the terminal 8 hydrogen atoms per unit cell with benzenesulfonic acid groups (─C_6_H_4_─SO_3_H). The nudged elastic band method^[^ ^46^ ^]^ was used to calculate the energy barriers for the penetration of proton, methanol, and formic acid.

## Conflict of Interest

The authors declare no conflict of interest.

## Author Contributions

Y.I. conceived the research idea. S.J. performed the experiments. S.J., T.N. analysed the samples T.O. performed the DFT calculations. S.J., T.O., T.N., Y.N., and Y.I. wrote the manuscript. J.F. contributed to the discussions and revisions.

## Supporting information

Supporting Information

## Data Availability

The data that support the findings of this study are available from the corresponding author upon reasonable request.
