# Supplementary material for: Suppression of Methanol and Formate Crossover through Sulfanilic‐Functionalized Holey Graphene as Proton Exchange Membranes
Source: Adv Sci (Weinh). 2023 Sep 8;10(31):2304082. doi: 10.1002/advs.202304082 (PMC10625063; doi:10.1002/advs.202304082)
Supplement: Supplementary file 1 — Supporting Information [file ADVS-10-2304082-s001.pdf]

ADVANCED SCIENCE
Open Access

Supporting Information

for *Adv. Sci.*, DOI 10.1002/advs.202304082

Suppression of Methanol and Formate Crossover through Sulfanilic-Functionalized Holey Graphene as Proton Exchange Membranes

Samuel Jeong, Tatsuhiko Ohto, Tomohiko Nishiuchi, Yuki Nagata, Jun-ichi Fujita and Yoshikazu Ito**

Supporting Information

Suppression of Methanol and Formate Crossover through Sulfanilic-functionalized Holey Graphene as Proton Exchange Membranes

Samuel Jeong,[1] Tatsuhiko Ohto,[2,3] Tomohiko Nishiuchi,[4] Yuki Nagata,[5] Jun-ichi Fujita,[1] and Yoshikazu Ito[1,*]*

[1]Institute of Applied Physics, Graduate School of Pure and Applied Sciences, University of Tsukuba, 1-1-1 Tennodai, Tsukuba, Ibaraki 305-8571, Japan

[2]Department of Materials Design Innovation Engineering, Nagoya University, Furo-cho, Chikusa-ku, Aichi 464-8603, Japan

[3]Graduate School of Engineering Science, Osaka University, 1-3 Machikaneyama, Toyonaka, Osaka 560-8531, Japan

[4]Department of Chemistry, Graduate School of Science, Osaka University, 1-1 Machikaneyama, Toyonaka, Osaka 560-0043, Japan

[5]Max Planck Institute for Polymer Research, Ackermannweg 10, 55128 Mainz, Germany.

*Email: ito.yoshikazu.ga@u.tsukuba.ac.jp, ohto@nagoya-u.jp

KEYWORDS: proton exchange membrane, crossover, methanol, formic acid, permeability, functional group introduction

1. Materials and methods

1.1. Fabrication of a Si_3N_4 chip-supported Nafion membrane

Nafion solution (5 wt.%, Wako) was coated on a Cu foil with a spin coater (1000 rpm, 3 min). The resulting Cu foil was dissolved with 0.25 M $Fe(NO_3)_3$ solution at 30°C for 12 h, and then the ultrapure water was used to replace the $Fe(NO_3)_3$ solution several times to remove the residual ions. The ultrapure water was then replaced with 1.0 M HCl solution and the HCl solution was heated at 60 °C for 1 hour to remove the adhering/residual metal ions on the Nafion. After 1 hour, the solution was replaced with ultrapure water several times to remove the residual ions. Then, the resulting single Nafion sheet floating on the water surface was transferred to the center of a window-attached Si_3N_4 chip (window area: 20 μm × 20 μm), and they were heated at 130 °C for 2 hours to closely adhere to each other (**Figure. S6**). In addition, the two-layers stacked Nafion sheet (i.e., Nafion/Nafion abbreviated as N/N) was transferred onto a window-attached Si_3N_4 chip and used as a Nafion membrane for control experiments. Note that all Si_3N_4 chips used in our experiments were hydrophilized by oxygen plasma etching in advance.

1.2. Fabrication of a Si_3N_4 chip-supported Nafion/graphene/Nafion membrane

After the graphene growth on the Cu foils with a standard chemical vapor deposition (CVD) method, O_2 plasma treatments were operated to remove the graphene on one side of the Cu foil. Then, the Nafion solution (5 wt.%, Wako) was coated on the graphene on the other side of the Cu foil with a spin coater (1000 rpm, 3 min). The resulting Cu foil was dissolved with 0.25 M $Fe(NO_3)_3$ solution at 30°C for 12 h, and then the ultrapure water was used to replace the $Fe(NO_3)_3$ solution several times to remove the residual ions. The ultrapure water was replaced with 1.0 M HCl solution and then the HCl solution was heated at 60 °C for 1 hour to remove the adhering/residual metal ions on the graphene. After 1 hour, the solution was replaced with ultrapure water several times to remove the residual ions. The resulting monolayer graphene sheet (1GL) attached by the spin-coated Nation sheet (namely, N/1GL) floating on the water surface was transferred to a center of window-attached Si_3N_4 chip (window area: 20 μm × 20 μm) with the other spin-coated Nafion sheet attached in advance (**Section 1.1**), and they were heated at 130 °C for 2 hours to closely adhere each other (**Figure. S6**). Thus, we fabricated Nafion/1GL/Nafion membrane (namely, N/1GL/N) on the Si_3N_4 chip. [1]

For the bi-layer graphene membrane, an additional step was required: transfer an isolated Nafion/1GL onto 1GL/Cu foil. Then, the Nafion/two-layer-graphene (namely, N/2GL)on the Cu foil was obtained. The same procedure was repeated to produce more than tri-layer graphene membrane (namely, N/3GL). The resulting Cu foil was dissolved with 0.25 M $Fe(NO_3)_3$ solution at 30°C for 12 h, and then the ultrapure water was used to replace the $Fe(NO_3)_3$ solution several times to remove the residual ions. The ultrapure water was replaced with 1.0 M HCl solution and then the HCl solution was heated at 60 °C for 1 hour to remove the adhering/residual metal ions on the graphene. After 1 hour, the solution was replaced with

ultrapure water several times to remove the residual ions. The resulting bi-/tri-layer graphene sheet attached by the spin-coated Nation sheet floating on the water surface was transferred to a center of window-attached Si_3N_4 chip (window area: 20 μm × 20 μm) with the other spin-coated Nafion sheet attached in advance (**Section 1.1**), and they were heated at 130 °C for 2 hours to closely adhere each other (**Figure. S6**). Thus, we fabricated Nafion/2GL/Nafion, and Nafion/3GL/Nafion membrane (namely, N/2GL/N, and N/3GL/N, respectively) on the Si_3N_4 chip. Moreover, the Nafion/xhGL/Nafion and Nafion/xfGL/Nafion (namely, N/xhGL/N, and N/xfGL/N, respectively; x refers to the number of graphene layers) were similarly obtained by the exchange from a graphene to a holey graphene or a sulfanilic functionalized holey graphene in the process.

The Nafion/graphene/Nafion membrane on the Si_3N_4 chip was reinforced with a polyethylene terephthalate (PET, 3.0 cm × 3.0 cm, 75 μm thickness) sheet to fix it in the H-type cell as separating membranes. As shown in **Figure. S11**, a hole with a diameter of ~2 mm was cut in the center of PET sheet and the Si_3N_4 chip with the graphene membrane was fixed to the hole by an acidic-stable liquid gasket. Before all experiments, it was confirmed that the gasket completely prevented the leakage of electrolytes between the cell chambers.

2. Determination of methanol and formic acid crossover rate

2.1. ^{1}H NMR elucidation for the crossover rates of methanol and formic acid

The concentrations of methanol and formic acid in the electrolyte solution samples on the counter electrode chamber were determined using an NMR spectrometer (Bruker, AVANCENEO 700 MHz). [2, 3] The ^{1}H NMR spectra were measured with water suppression by the excitation sculpting method. For elucidation of the concentration of formate using the NMR technique, a standard curve calibration method between the relative integration area to the internal standard and the concentration of formic acid has been widely used[3] because the usual measurement parameters in ^{1}H NMR could not afford a proportional relationship owing to the difference of spin-lattice relaxation time T_1 between formate and internal standard, and a relatively long T_1 of formate. However, such a method is not quantitative, and the quantitative analysis of the formate concentration can be performed by the adequate setting of prescan delay (PD) time for affording a fully relaxed spin state of formate. In the measurement condition (H_2O : D_2O = 5 : 1 of 0.05 M H_2SO_4 at 298 K), the T_1 time of formate and dimethyl sulfoxide (DMSO) as the internal standard were determined at 9.0 sec. and 3.8 sec., respectively. [4] In addition, we evaluated the PD time dependency of the relative integration area for formate. According to the evaluation, the relative integration area for methanol and formate is almost saturated over 60 seconds of PD time. Therefore, we set the PD time to 60 s in our NMR measurements to detect the methanol and form.

2.2. Estimation of methanol and formate crossover rate by using GC

The concentrations of methanol and formic acid (formate) in the electrolyte solution samples were determined by gas chromatography (GC; Nexis GC-2030, Shimadzu). A flame ionization detector (FID) detector and a dielectric barrier discharge ionization detector (BID) were equipped on a GC to detect methanol and formic acid, respectively. In order to prevent the adsorption of formate on the injection port and inner wall in the column, phosphoric acid treatment to the glass insert and the column was carried out before the measurements (see more details in Shimazu application note: LAAN-A-GC-E044A or previous report[4]). Moreover, for the sample treatment, the obtained electrolyte solutions were injected into a cation-exchange cartridge (Maxi-clean 0.5 mL iC-Ba) to remove the sulfuric ion. Note that 0.5 M H_2SO_4 as electrolytes strongly disturb the detection of the target molecules. Then, the resulting solutions were injected into the GC. Using 1 ppm, 5 ppm, 10ppm, 50 ppm, and 100 ppm of methanol or formic acid (diluting the HPLC grade methanol or formic acid by ultrapure water) as standard curves, the concentrations of methanol and formic acid were estimated.

2.3. Standard curve of methanol for determination of crossover rate

1 ppm, 5 ppm, 10 ppm, and 50 ppm methanol in ultrapure water were similarly measured three times. The peaks showed good reproducibility. Using the averaged peak area, we plotted the standard curve (**Figure. S15**). Then, we analyzed the electrolyte solutions by GC and estimated the concentration of methanol from the peak area. [4, 5]

2.4. Standard curve of formate for determination of crossover rate

1 ppm, 10 ppm, 50 ppm, and 100 ppm formic acid in ultrapure water were similarly measured three times under the phosphoric acid treatment conditions. The peaks showed good reproducibility. Using the averaged peak area, we plotted the standard curve (**Figure. S15**). Then, we analyzed the electrolyte solutions by GC and estimated the concentration of formate from the peak area. [4, 5]

3. Supporting discussion

3.1. Characterizations of graphene membranes

The graphenes were characterized by transmission electron microscopy (TEM), scanning transmission electron microscopy (STEM), X-ray photoelectron spectroscopy (XPS), and Raman spectroscopy. TEM and STEM images indicated that the hGL and fGL without any Nafion sheets showed 2−20 nm holes (**Figure. 1a, b**, and **Figure. S2, S4**). The area of the hole regions was about 10% of the entire graphene sheet, and the average size of the holes was about 5 nm (**Figure. S4**). The distribution of S and O element from sulfanilic functional groups were obtained by EDS for fGL. The S and O element distribution were high in the hole regions of the graphene, which indicated that the introduced functional groups existed mainly in the hole areas (**Figure. 2c-e**). Raman mapping images of N/1GL/N, and N/1hGL/N on the window-attached Si_3N_4 chip (widow size: 20 × 20 μm) indicated high 2D-band to G-band (I_{2D}/I_G) intensity ratios of 1.98 to 4.00, suggesting that the single graphene layer was highly crystalline (**Figure. S3**). The I_{2D}/I_G ratio of N/1fGL/N was lower than that of N/1GL/N and N/1hGL/N due to the partial graphene oxide-like lattices caused by the increase in oxygen bonds during the functional group modification. [6] This was consistent with the broadening of C 1s peaks with the enhancement of oxidized carbon peaks (C=O and C-O) on the XPS results (**Figure. S5**). Moreover, Raman mapping images of the N/1GL/N showed low D-band to G-band (I_D/I_G) intensity ratios of 0.03−0.07. In contrast, the N/1hGL/N exhibited relatively high I_D/I_G ratios of 0.46−0.78 (**Figure. S3**), which suggested that many defects were induced in the hole region. In addition, the N/1fGL/N showed higher I_D/I_G ratios of 0.88−1.75 than the N/1hGL/N, which indicated that the sulfanilic functional group induced more defects in the N/1hGL/N. As the number of graphene layers was increased from mono- to bi- or tri-layers, the I_{2D}/I_G ratios were decreased to approximately 1.02−2.04 for N/2GL/N, N/2hGL/N, and N/2fGL/N, and 0.37−1.72 for N/3GL/N, N/3hGL/N, and N/3fGL/N (**Figure. 2c-d**, **Figure S9** and **10**).

3.2. Proton penetration and fuel crossover test of bi-layer and tri-layer graphene

The areal proton conductivity, crossover rates of methanol and formate for N/GLs/N, N/hGLs/N, and N/fGLs/N were systematically investigated to understand the stacking effects of multi-layer graphene (**Figure. 3c**, **Figure. 4**, **Figure S13, S16,** and **Table. S1, S2**). In the bi-layer graphene membrane, the areal proton conductivity of N/2GL/N (0.034 mS cm^{-2}) was lower than that of N/1GL/N (0.135 mS cm^{-2}). The areal proton conductivities of N/2hGL/N (801 mS cm^{-2}) and N/2fGL/N (1232 mS cm^{-2}) decreased by 24.5% and 17.9% in comparison to the N/1hGL/N (1060 mS cm^{-2}) and N/1fGL/N (1500 mS cm^{-2}), respectively, due to the blocking of proton penetration between the interlayer of graphenes. The crossover rates of methanol were 0.143, 0.290, and 0.169 mol $m^{-2}s^{-1}$ for the N/2GL/N, the N/2hGL/N, and the N/2fGL/N, respectively, and the crossover rates of formate were 0.065, 0.130, and 0.117 mol $m^{-2}s^{-1}$ for the N/2GL/N, the N/2hGL/N, and the N/2fGL/N, respectively. The crossover rates

of N/2fGL/N decreased by 70.5 % for methanol and 28.3% for formate in comparison to that of N/1fGL/N (0.573 mol $m^{-2}s^{-1}$ for methanol, and 0.163 mol $m^{-2}s^{-1}$ for formate). Moreover, in the tri-layer graphenes, the N/3fGL/N showed the highest proton conductivity (818 mS cm^{-2}) among the N/3GL/N (0.016 mS cm^{-2}), the N/3hGL/N (509 mS cm^{-2}) and the N/3fGL/N, which was almost half of the proton conductivity of Nafion membrane (N/N of 1930 mS cm^{-2}) (**Table. S1**). The N/3fGL/N showed a lower methanol crossover rate (0.120 mol $m^{-2}s^{-1}$) and formate crossover rate (0.052 mol $m^{-2}s^{-1}$) than those of N/3hGL/N (0.218 and 0.097 mol $m^{-2}s^{-1}$ for methanol and formate). Their values were close to the methanol and formate crossover rate of N/3GL/N (0.095 and 0.039 mol $m^{-2}s^{-1}$ for methanol and formate, respectively).

3.3. Performance comparison for areal proton conductivity and methanol/formate crossover rate

In general, a membrane which has higher proton conductivity and the lower fuel crossover is best. Since the Nafion membrane is widely used as a benchmark for proton exchange membranes, the membrane performance previously reported could be compared based on the following simple relationship (**Figure. 4**).

$$\boldsymbol{MP}\ (\textbf{membrane performance}) = +\log\frac{\sigma}{\sigma_{Nafion}} - \log\frac{\mu}{\mu_{Nafion}} \qquad \cdots\cdots (\textbf{S1})$$

where σ was the areal proton conductivity (mS cm^{-2}), and μ was the methanol/formate crossover rate (mol $m^{-2}s^{-1}$).

Based on the above relationship, the *MP* values for the various graphene membranes used in our study and the reported proton exchange membranes were calculated for methanol and formate (**Table. S3** and **S4**). Note that reported proton conductivities used here have different physical units such as proton conductivity and areal proton conductivity. In other words, the analysis method has not been unified in this field. Thus, we compared the degree of proton conductivity of each sample membrane by using a ratio of the proton conductivity of each sample membrane divided by that of the Nafion membrane (reference) in the same report. In addition, the methanol crossover was also evaluated with different physical units by reports such as crossover rate (mol $m^{-2}s^{-1}$), permeability ($cm^{-2}s^{-1}$), and crossover current density (A cm^{-2}). Thus, we similarly compared the degree of crossover rate of each sample membrane including the physical unit by using a ratio of the crossover amount of each sample membrane divided by that of the Nafion membrane (reference) in the same report. For the formic acid, we only compared the crossover ratio with our samples because there were no reports about formic acid crossover reported with (areal) proton conductivity of both the sample membrane and the Nafion membrane. The larger positive value of *MP* indicated a better balance between areal proton conductivity and crossover suppression ratio. In the case of the proton conductivity of

sample membrane normalized by the proton conductivity of Nafion vs. The methanol crossover rate of sample membrane normalized by the methanol crossover rate of Nafion, the N/2fGL/N or N/3fGL/N showed the best balance (**Figure. 4a**). In the case of the proton conductivity of sample membrane normalized by the proton conductivity of Nafion vs. the formate crossover ratio of sample membrane normalized by the formate crossover ratio of Nafion, the N/3fGL/N showed the best balance (**Figure. 4b**).

3.4. Electrochemical measurements using industrial Nafion® (117 and 212) membrane with/without 3fGL

Nafion thickness dependence of areal proton conductivity and methanol/formate crossover rates were investigated by using 3fGL-pasted Nafion®117 (thickness: 180 μm) and Nafion®212 (thickness: 50 μm). N/3fGL/Nafion®117 and N/3fGL/Nafion®212 were similarly prepared with an exchange of the other spin-coated Nafion to Nafion®117 or Nafion®212 (**Figure. S6**). Firstly, we spin-coated (1000 rpm, 3 min) Nafion solution to a 1fGL on a Cu foil. The resulting Cu foil was dissolved with 0.25 M $Fe(NO_3)_3$ solution at 30°C for 12 h, and then the ultrapure water was used to replace the $Fe(NO_3)_3$ solution several times to remove the residual ions. The ultrapure water was replaced with 1.0 M HCl solution and then the HCl solution was heated at 60 °C for 1 hour to remove the adhering/residual metal ions on the graphene. After 1 hour, the solution was replaced with ultrapure water several times to remove the residual ions. Then, transfer an isolated N/1fGL onto 1fGL/Cu foil then heated and dried at 120°C. Then, the Nafion/two-layer-functionalized graphene (namely, N/2fGL)on the Cu foil was obtained. The same procedure is used to remove the Cu foil and transfer N/2fGL to 1fGL on the copper foil and heated and dried. The copper foil is then removed again and N/3fGL is isolated. The resulting Nafion-coated 3fGL (N/3fGL) floating on the water surface was transferred to the center of Nafion®117 or Nafion®212 (area: 3 mm in diameter) (**Figure. S6**). The obtained Nafion/3fGL/Nafion®117 and Nafion/3fGL/Nafion®212 were called as N/3fGL/Nafion®117 and N/3fGL/Nafion®212 in main text, respectively. The N/3fGL/Nafion®117 and N/3fGL/Nafion®212 were reinforced with a polyethylene terephthalate (PET, 3.0 cm × 3.0 cm, 75 μm thickness) sheet to fix it in the H-type cell as separating membranes. As shown in **Figure. S11**, a hole with a diameter of ~2 mm was cut in the center of PET sheet and the N/3fGL/Nafion®117 or N/3fGL/Nafion®212 was fixed to the hole by an acidic-stable liquid gasket. The electrolyte contact area on the membrane was further controlled to be 0.2 mm in diameter with an acidic-stable liquid gasket, and the membrane window area was precisely measured with an optical microscope for the normalization of proton conductivity. Before all experiments, it was confirmed that the gasket completely prevented the leakage of electrolytes between the cell chambers. Thereafter, areal proton conductivity and methanol/formate crossover tests were conducted with the same procedures.

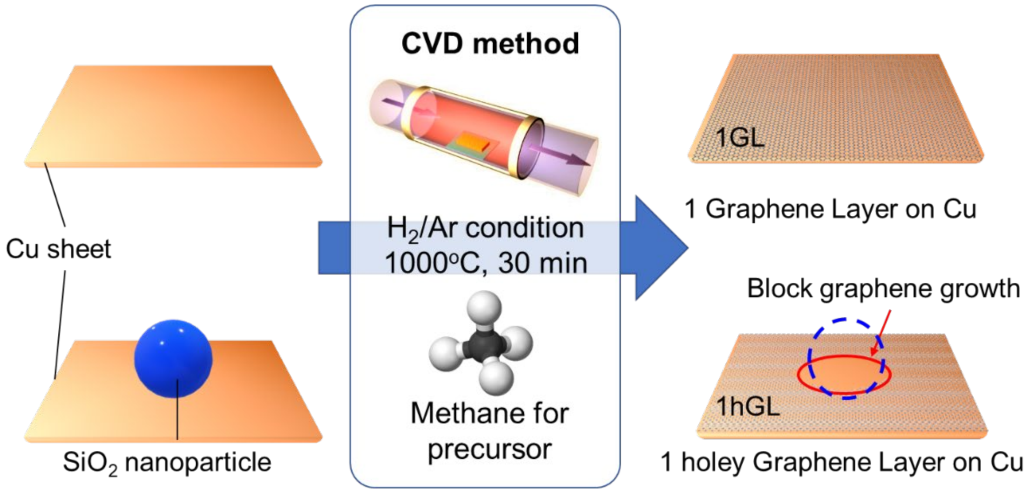

Figure. S1. Schematic illustrations of graphene and holey graphene synthesis.

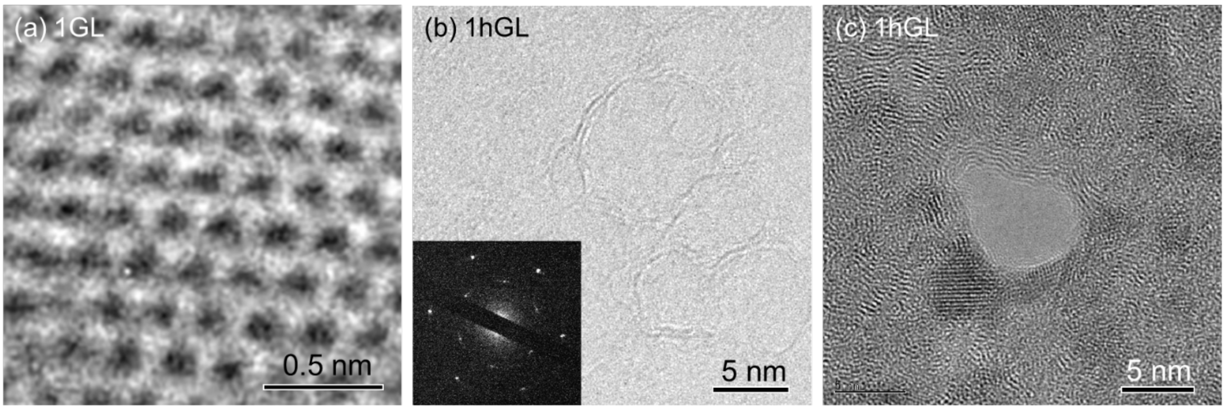

Figure. S2. TEM images of (a) 1GL and (b-c) 1hGL. The inset of (b) shows electron diffraction patterns.

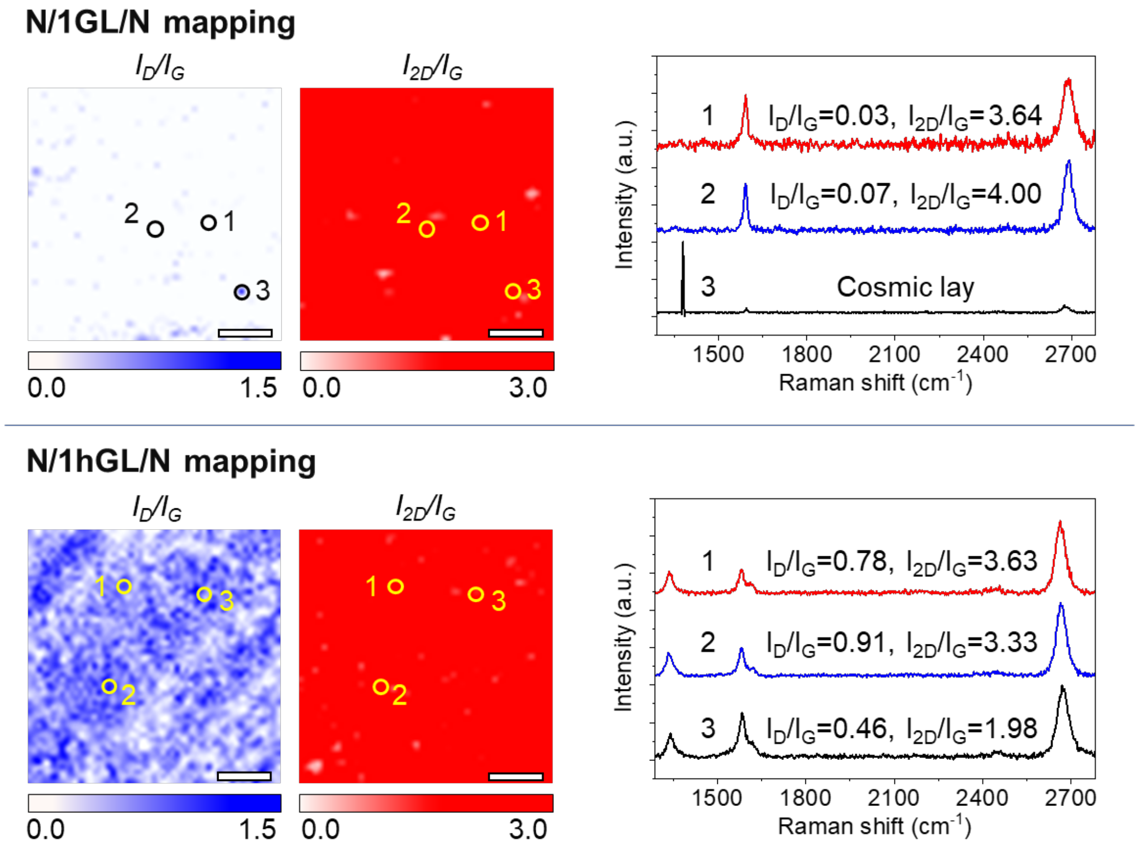

Figure. S3. Raman mapping images of N/1GL/N and N/1hGL/N on a window-attached Si_3N_4 chip (left side). Raman spectrums (right side) were collected at the 1, 2, and 3 points indicated on the corresponding images on the left side. The scale bar was 5 µm, and the image size was 20.8 µm × 20.8 µm.

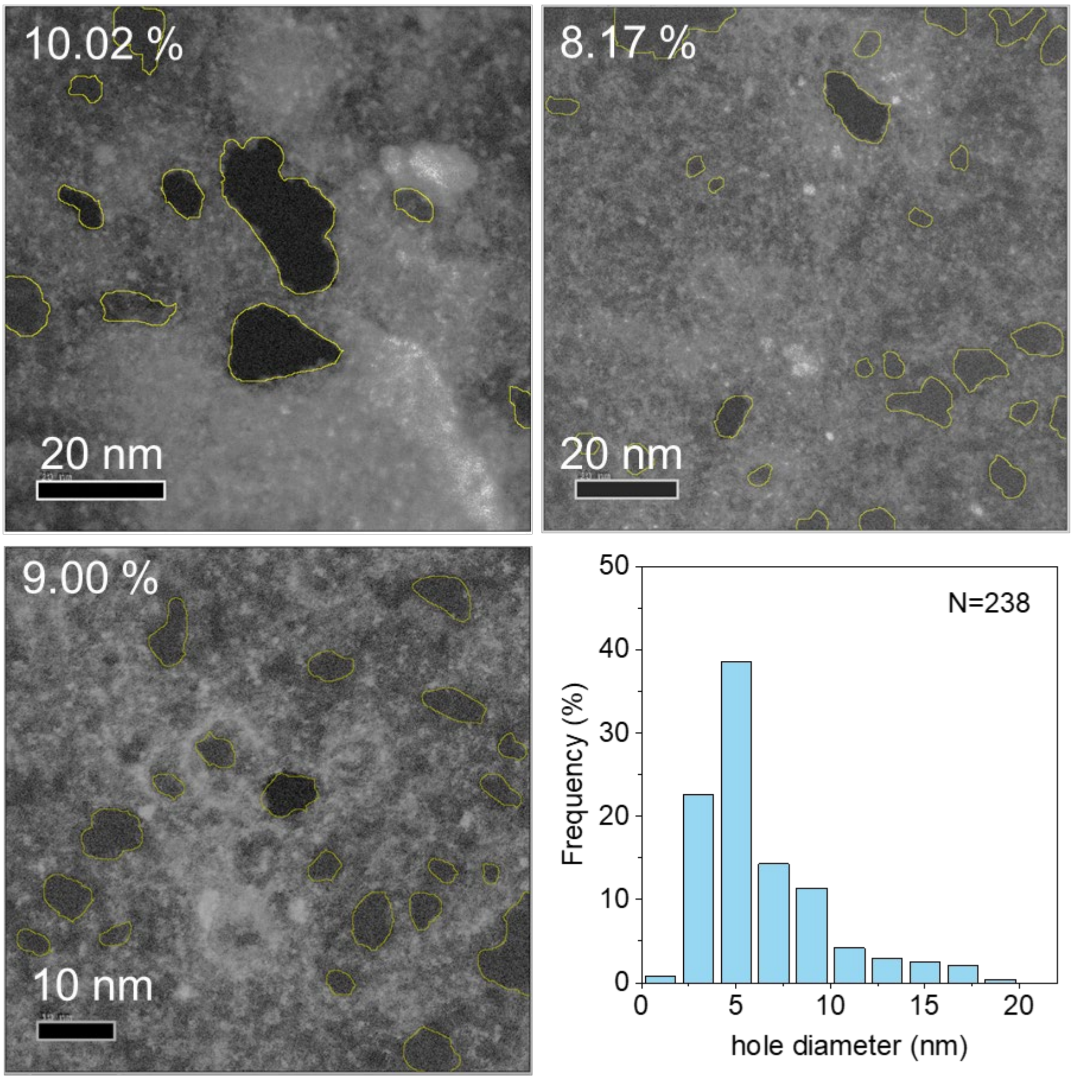

Figure. S4. Estimation of hole/non-hole region area ratio on the 1hGL, and the frequency distribution of hole diameter on the graphene.

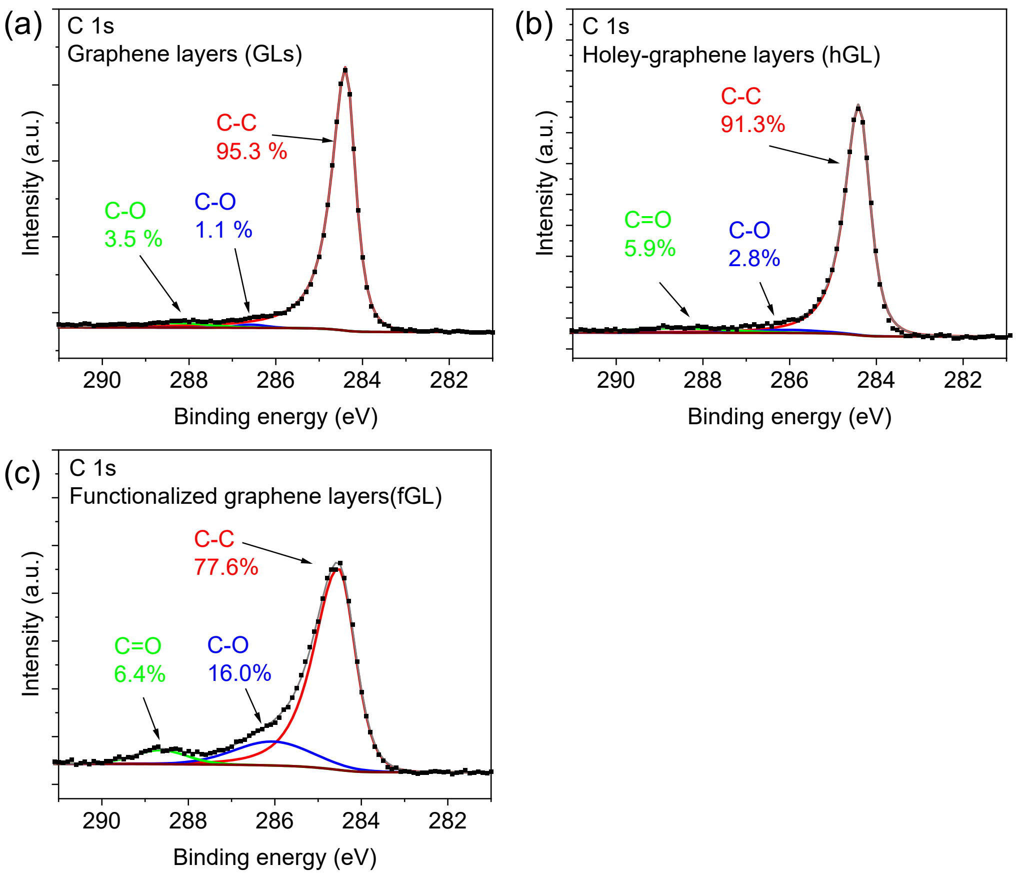

Figure. S5. XPS C1s spectra for (a) GL, (b) hGL, and (c) fGL.

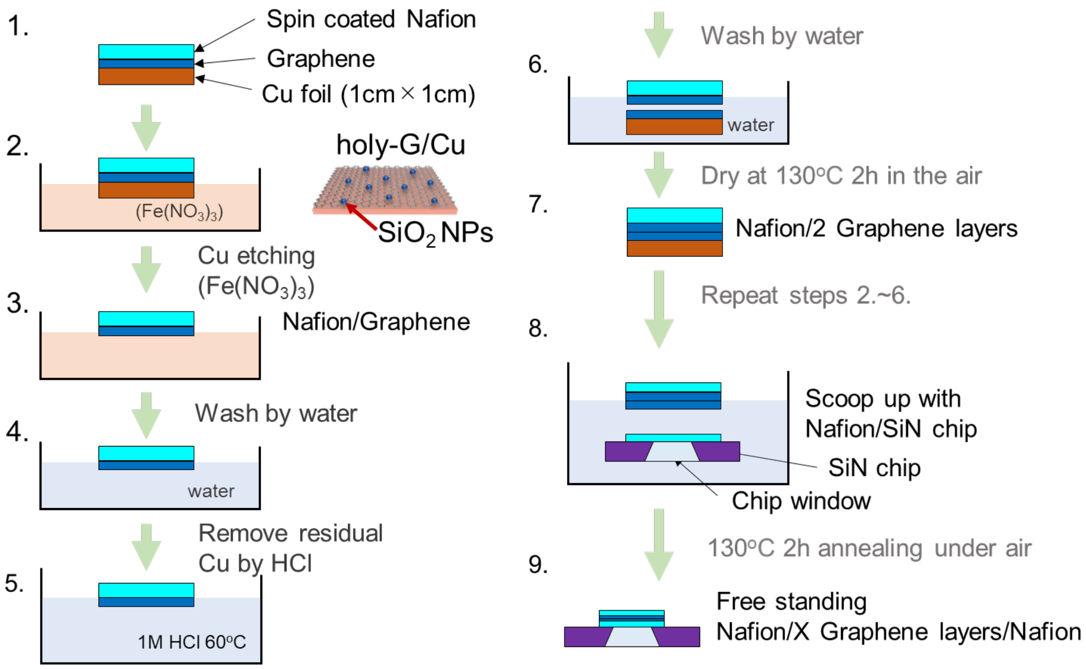

Figure. S6. Fabrication of Nafion/graphene/Nafion membrane on a Si_3N_4 chip. For preparing the membrane with multilayer graphene ("X" presented the number of graphene layers in the figure), the processes from steps 2 to 7 were simply repeated.

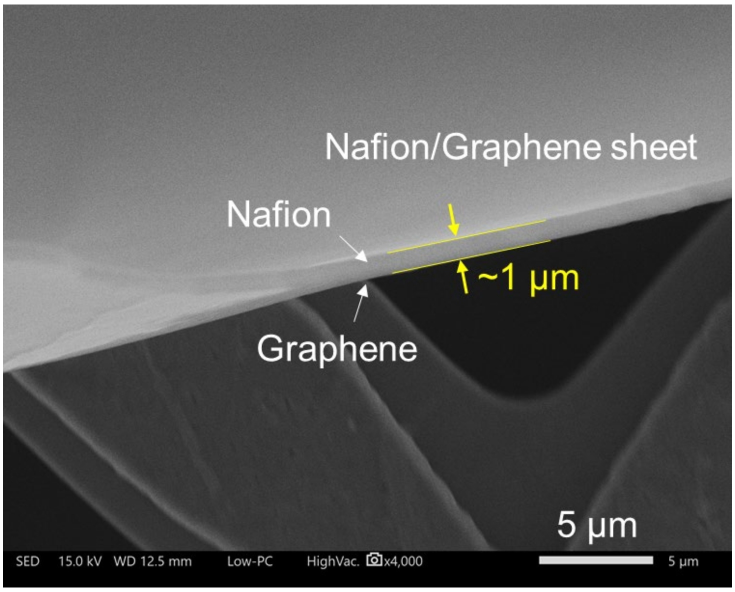

Figure. S7. SEM image of isolated Nafion/graphene membrane. A spin-coated Nafion sheet on the graphene was ~1 μm thick. The Nafion/graphene/Nafion membrane was ~2 μm.

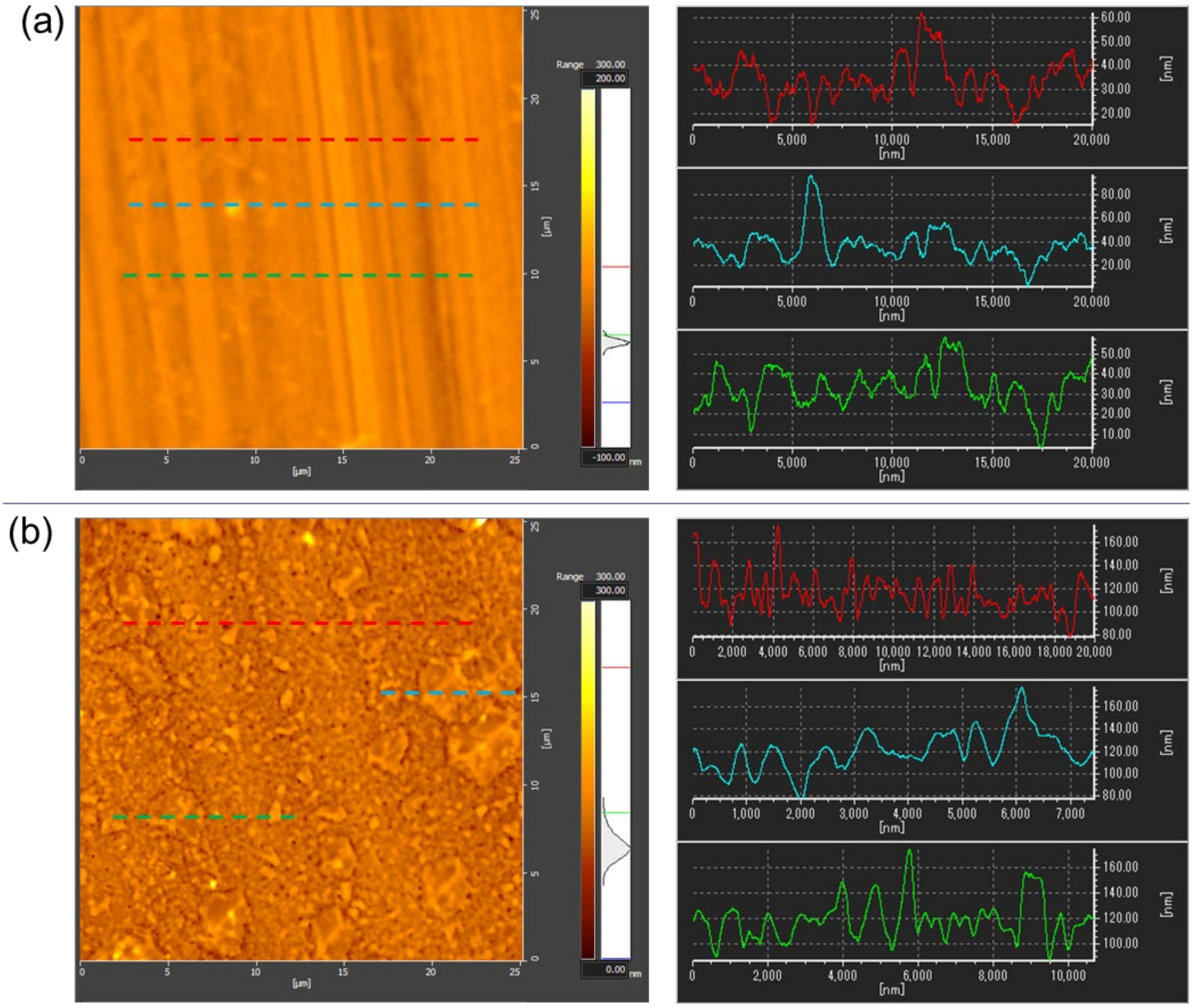

Figure. S8. Typical AFM images of (a) Nafion/Nafion (N/N), and (b) Nafion sandwiched 3fGL (N/3fGL/N). Line profiles (right side) were collected at each line indicated on the corresponding AFM images on the left side. The maximum roughness was 40 nm, and 80 nm for N/N and N/3fGL/N, respectively.

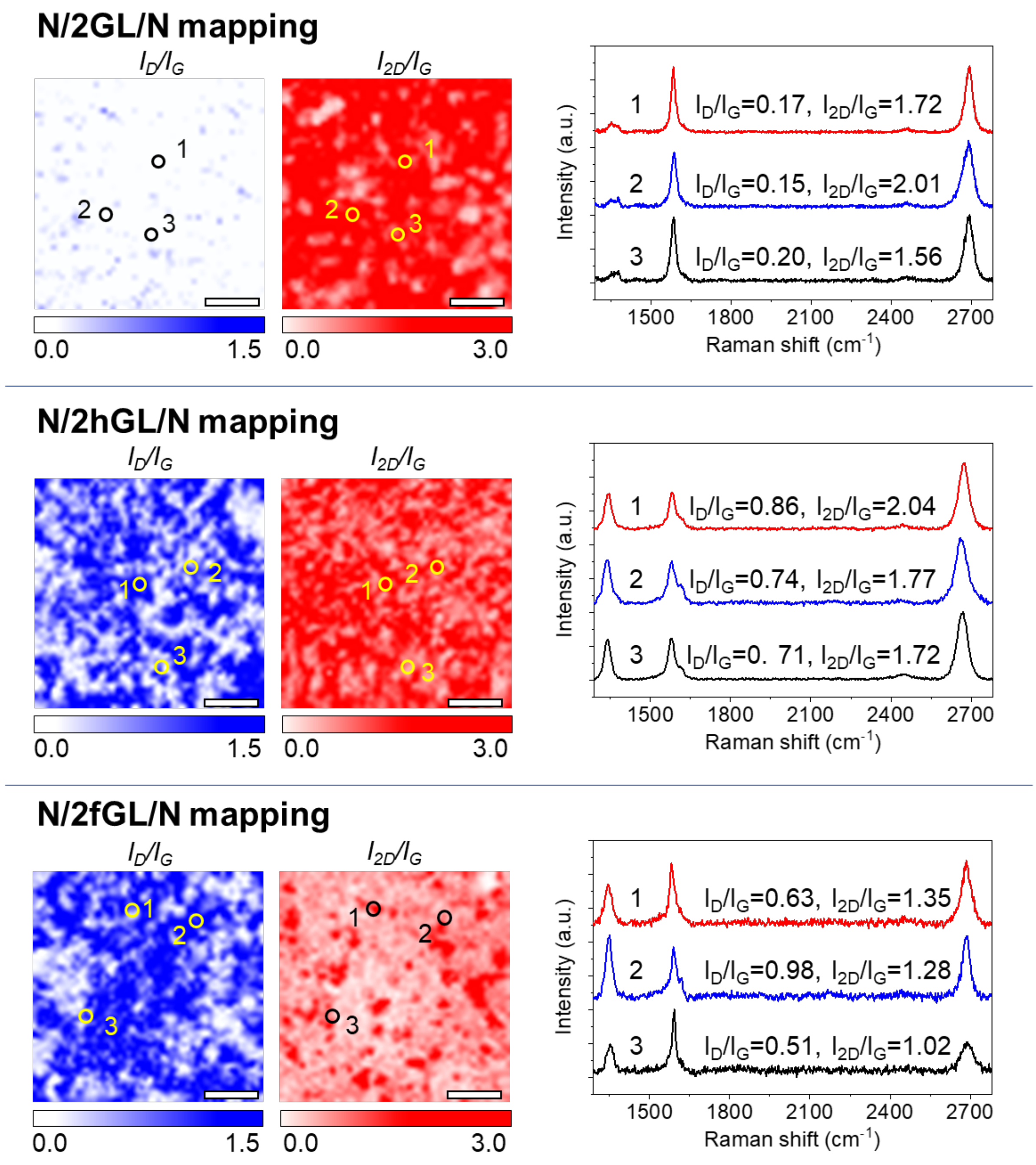

Figure. S9. Raman mapping images of N/2GL/N, N/2hGL/N, and N/2fGL/N on a window-attached Si_3N_4 chip (left side). Raman spectrums (right side) were collected at the 1, 2, and 3 points indicated on the corresponding mapping images on the left side. The scale bar was 5 μm, and the image size was 20.8 μm × 20.8 μm.

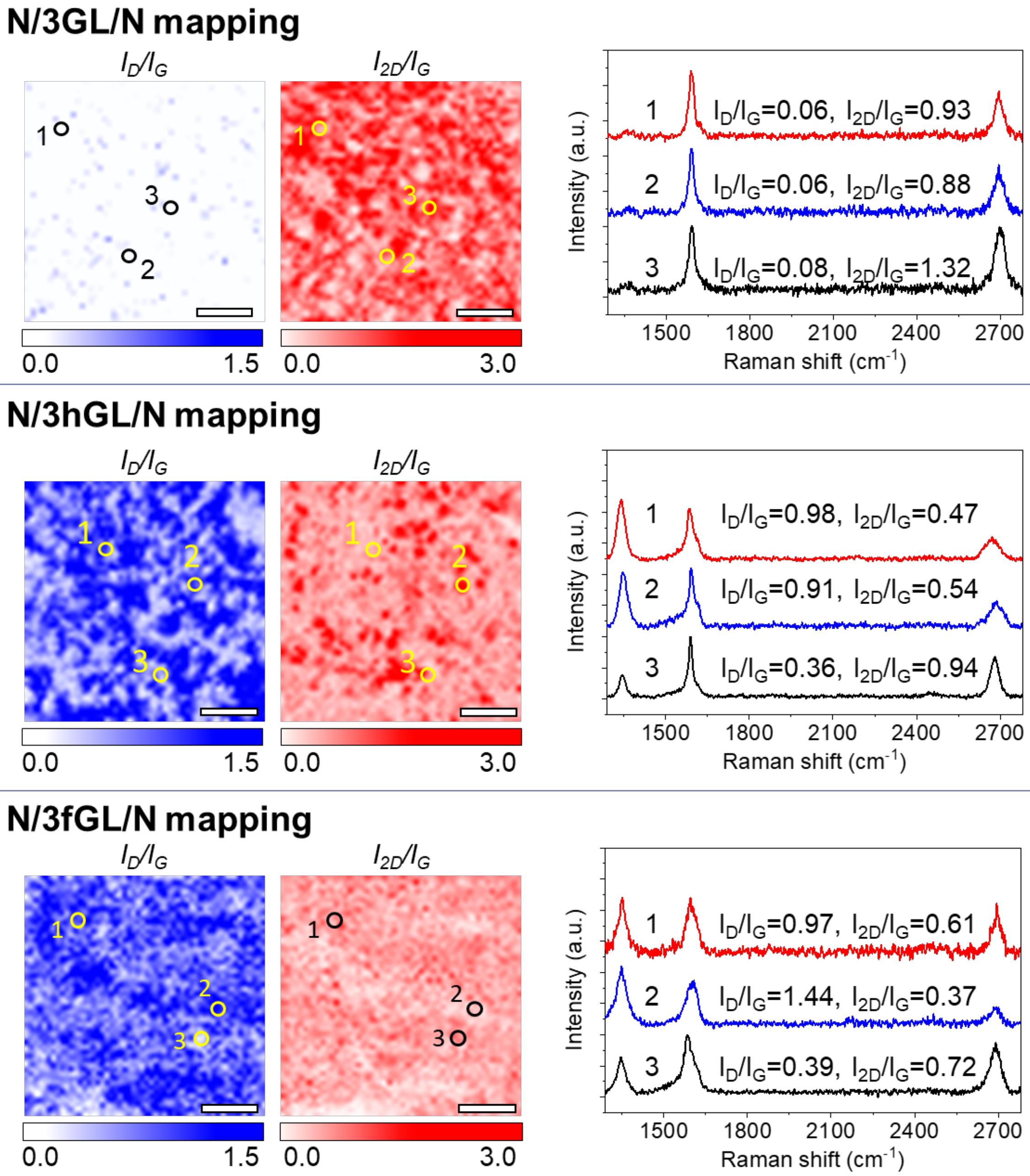

Figure. S10. Raman mapping images of N/3GL/N, N/3hGL/N, and N/3fGL/N on a window-attached Si_3N_4 chip (left side). Raman spectrums (right side) were collected at the 1, 2, and 3 points indicated on the corresponding mapping images on the left side. The scale bar was 5 µm, and the image size was 20.8 µm × 20.8 µm.

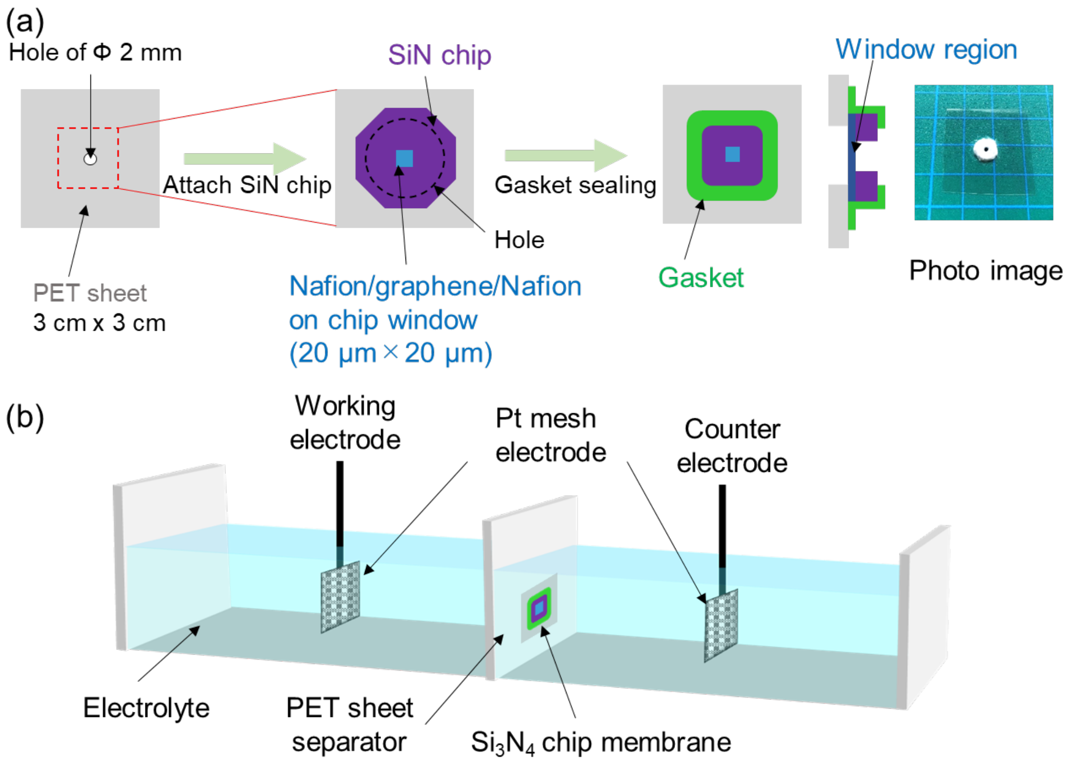

Figure. S11. Membrane device fabrications for a H-type cell. (a) Preparation of PET sheet attached membrane chip for the H-type cell. (b) Schematic illustration of the H-type cell. 0.05 M H_2SO_4 electrolyte was carefully filled in both the working electrode and counter electrode chambers. Pt-mesh electrodes were used for both electrodes. The distance between the Pt-mesh electrodes was fixed as 2 cm.

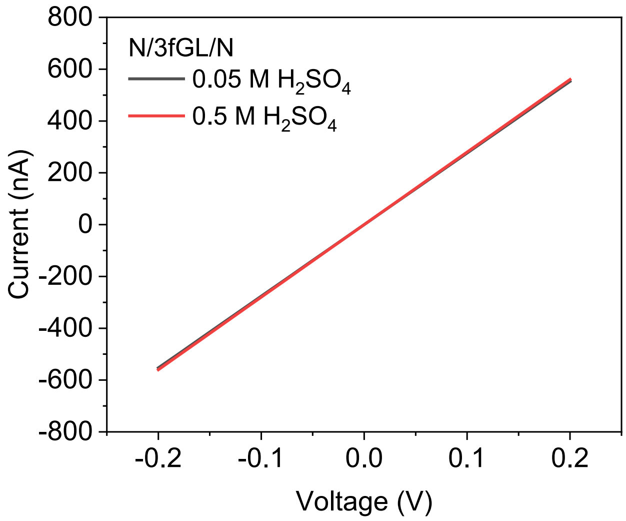

Figure. S12. Current-voltage (*I-V*) characteristics of N/3fGL/N for the electrolyte concentration dependence of 0.5 M and 0.05 M H_2SO_4.

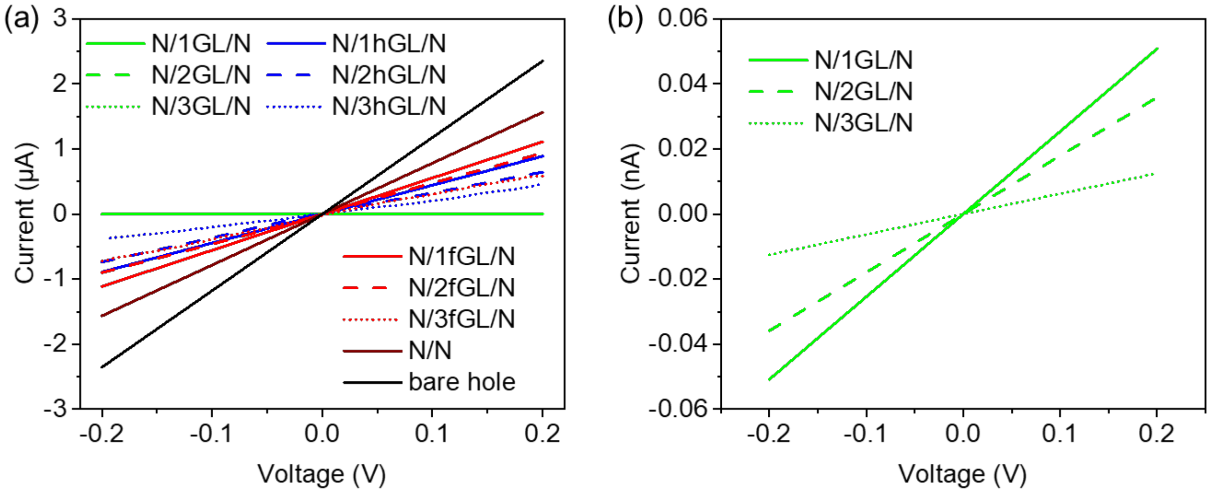

Figure. S13. (a) *I-V* characteristics of proton current through various graphene membranes in 0.05 M H_2SO_4 electrolyte. (b) Enlarged plots for N/xGL/N (x refers to the number of graphene layers).

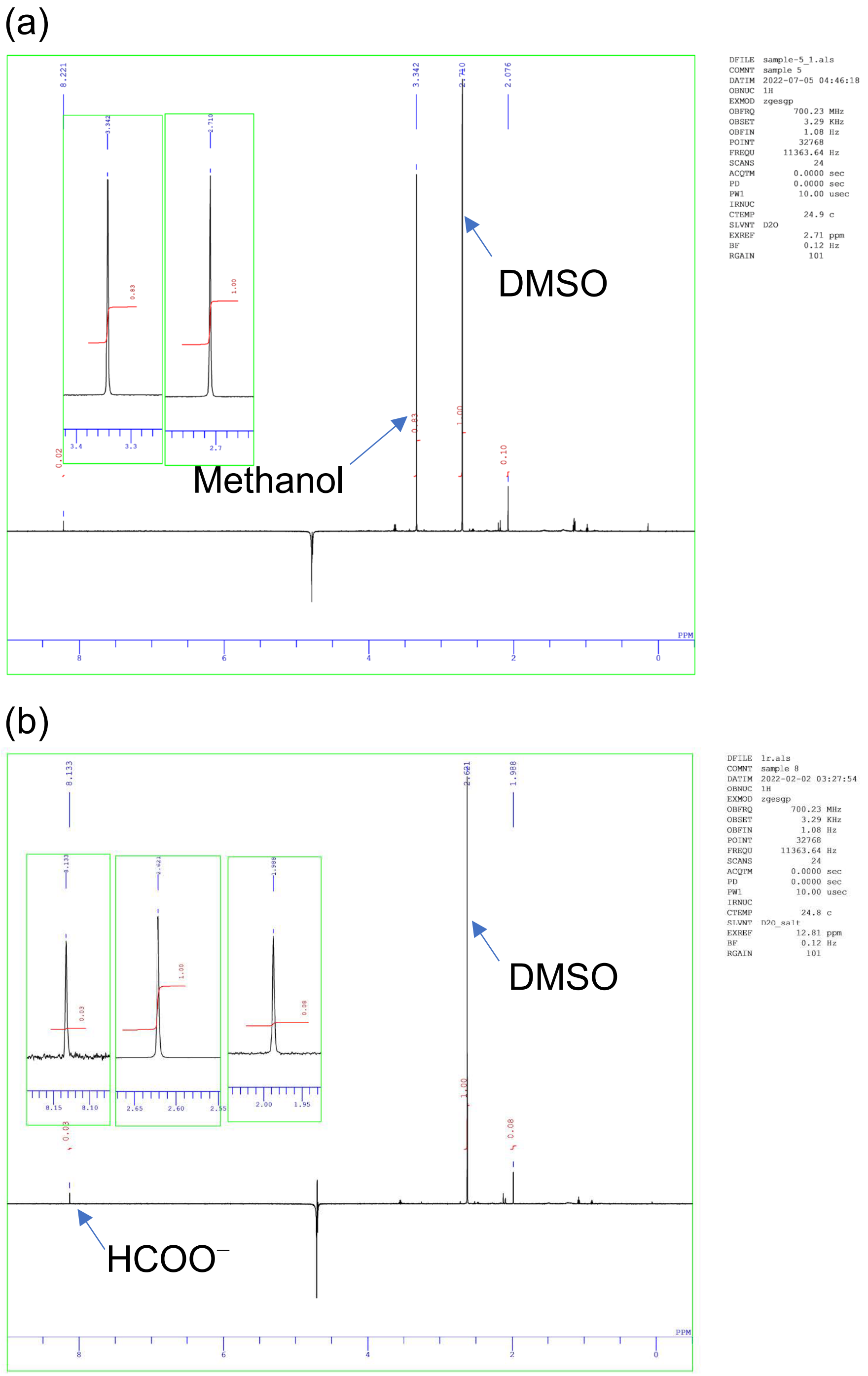

Figure. S14. Typical NMR charts of (a) methanol and (b) formate after the 5 h crossover tests.

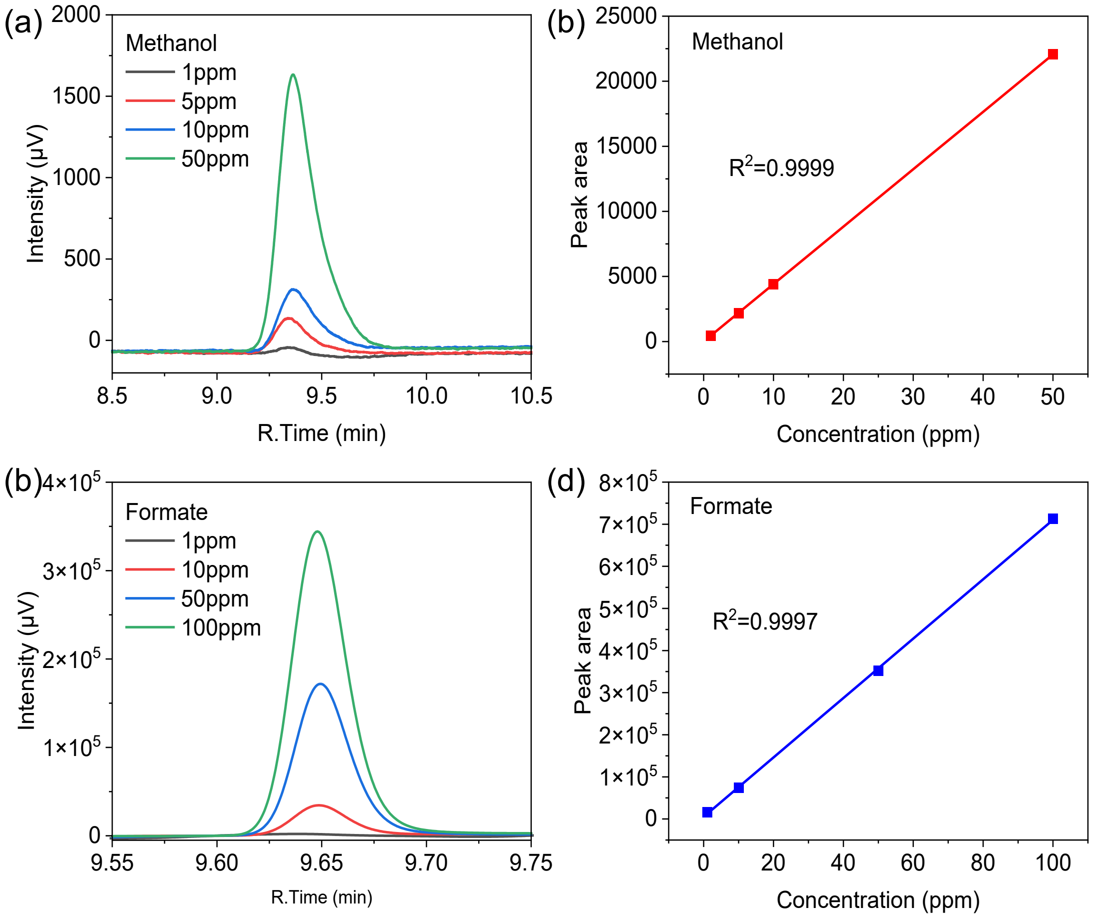

Figure. S15. (a) Gas chromatograms of standard methanol samples. (b) Methanol calibration curve obtained from the gas chromatograms peak areas. (c) Gas chromatograms of standard formate samples. (d) Formate calibration curve obtained from the gas chromatograms peak areas.

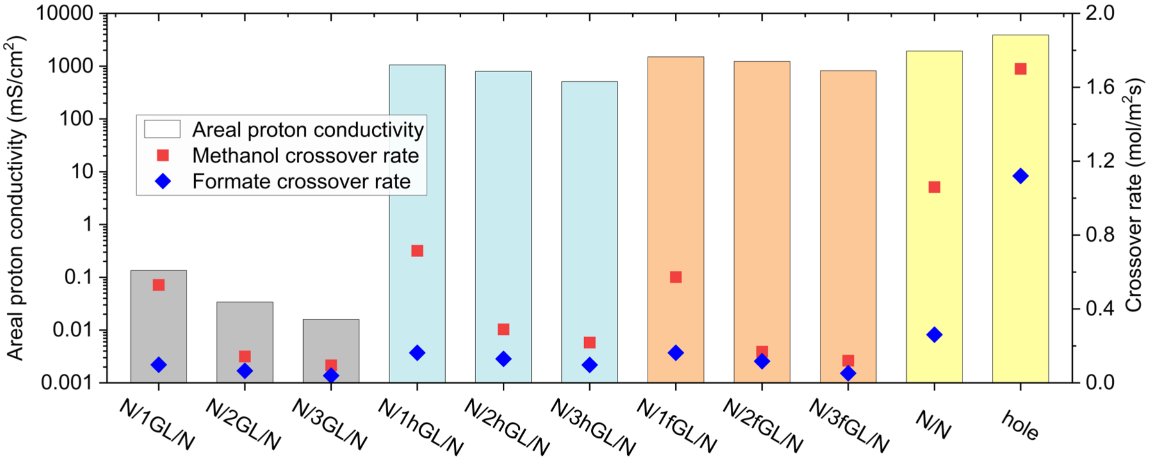

Figure. S16. Summary of areal proton conductivities and crossover rates of methanol and formate through the various graphene membranes and a Nafion membrane. “hole” represented the data without any membranes (i.e. control experiments). The left y-axis presented the areal proton conductivity from *I-V* characteristics and the right y-axis presented the crossover rate which indicated methanol and formate crossover molar per unit area and time.

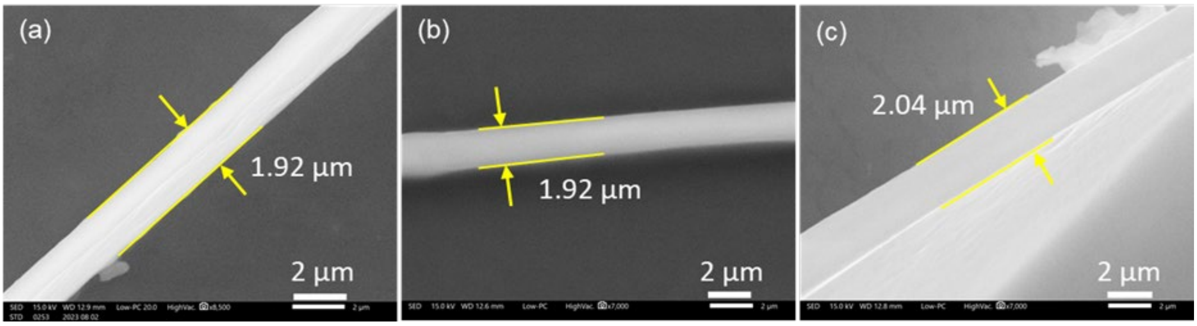

Figure. S17. Cross-sectional scanning electron microscopy images of N/3fGL/N after (a) *I-V* test, (b) methanol, and (c) formate crossover test.

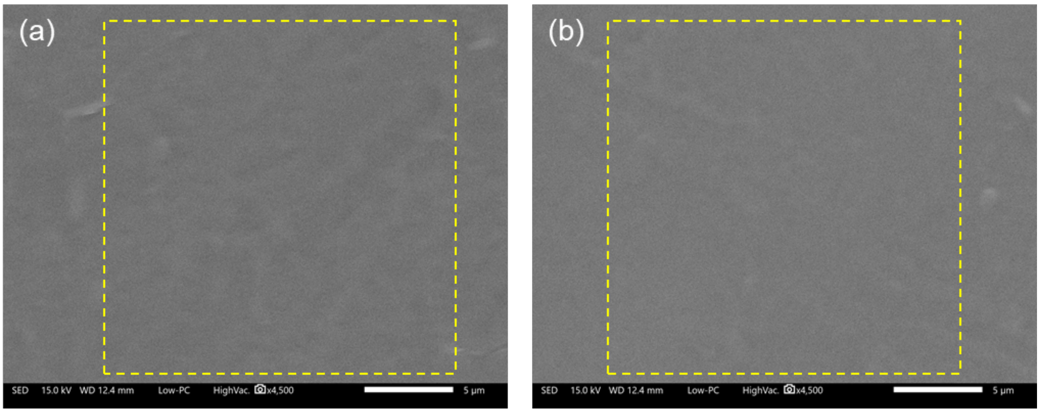

Figure. S18. Scanning electron microscopy images of N/3fGL/N after (a) methanol, and (b) formate crossover test.

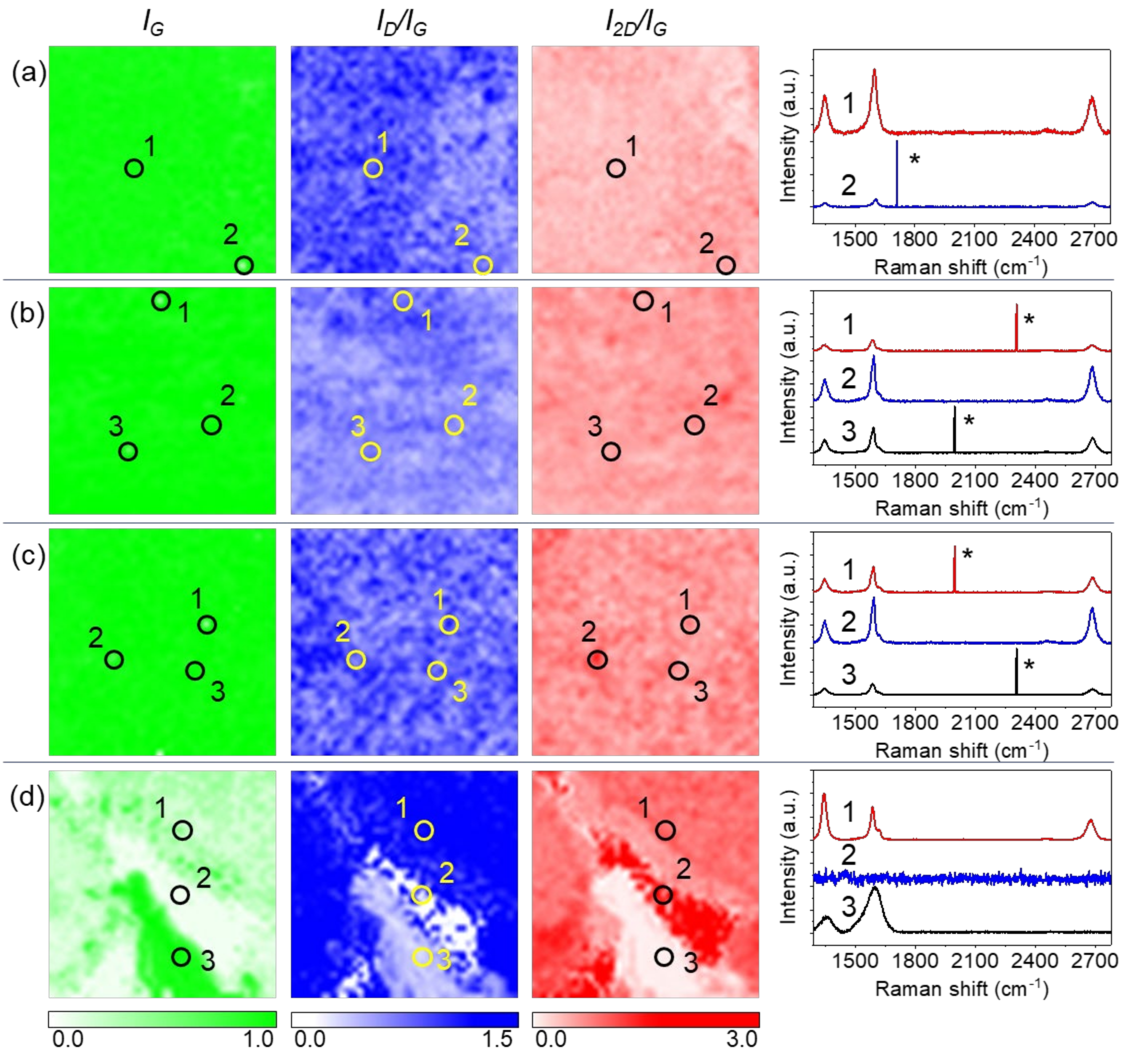

Figure. S19. Raman mapping images of N/3fGL/N on a window-attached Si_3N_4 chip. The I_G, I_D/I_G, and I_{2D}/I_G mapping (a) before the test, after (b) methanol, and (c) formate crossover test. (d) Example of a peeled graphene membrane. Representative Raman spectrums were collected at the 1, 2, and 3 points in the corresponding mapping data. The asterisk, *, in the spectra indicated the cosmic ray peak. The image size was 22 µm × 22 µm.

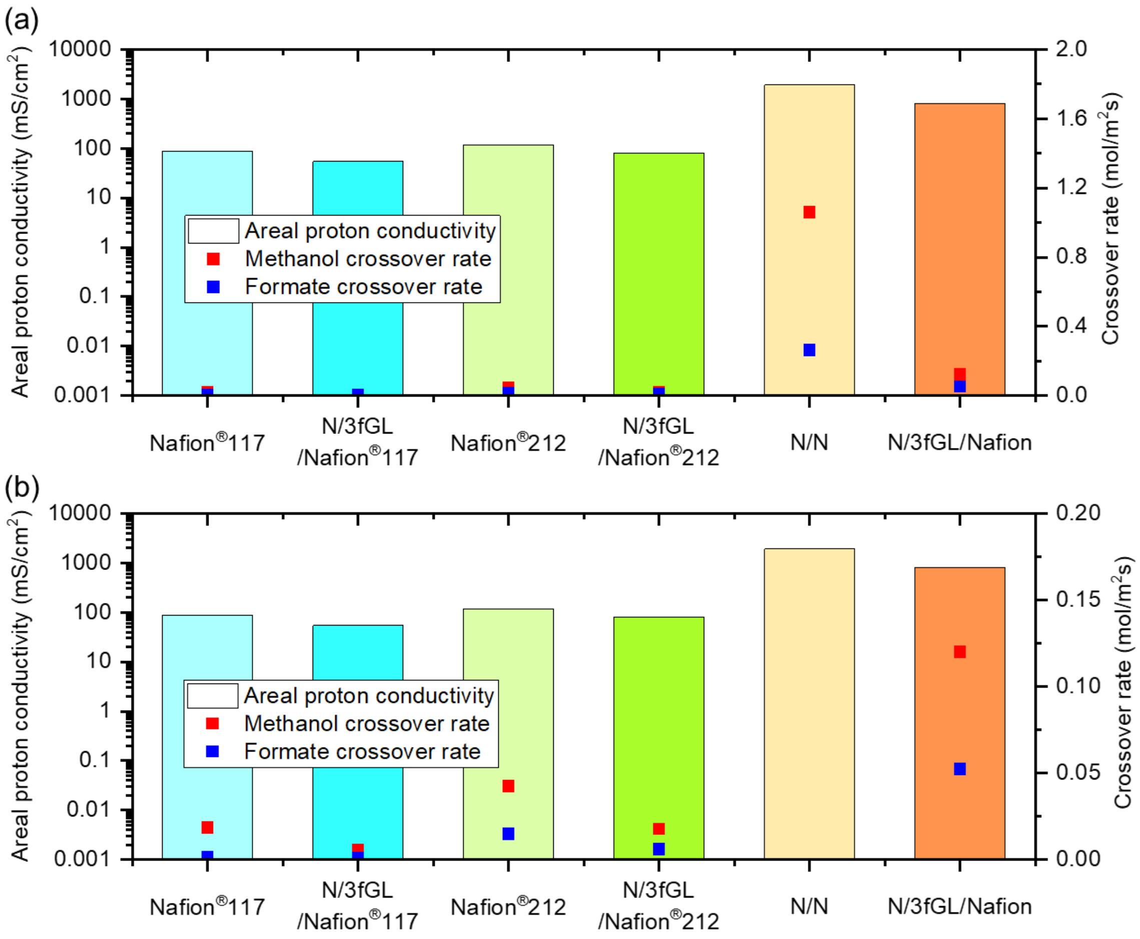

Figure. S20. Summary of areal proton conductivities and crossover rates of methanol and formate with/without graphene membranes on various Nafion membranes. (b) Zoom in of Figure (a). The 'N' is the Nafion we made from the Nafion solution described in the manuscript.

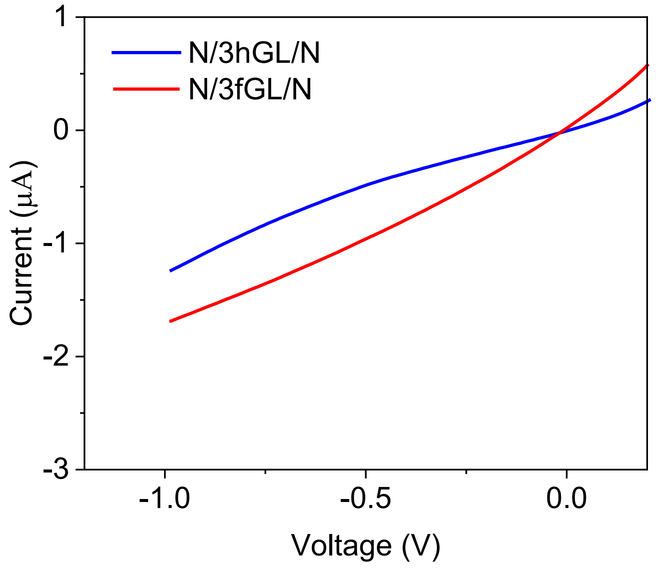

Figure. S21. *I-V* characteristics of proton penetration through N/3hGL/N and N/3fGL/N in the range from −1.00 to + 0.20 V.

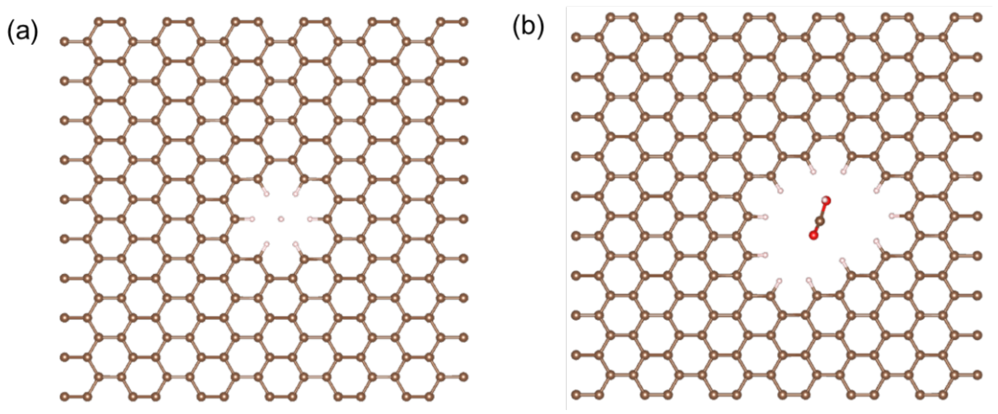

Figure. S22. DFT models of a proton, and formic acid passage through the hole of holey graphene. (a) hole size of ϕ 0.3 nm, and (b) hole size of ϕ 1 nm.

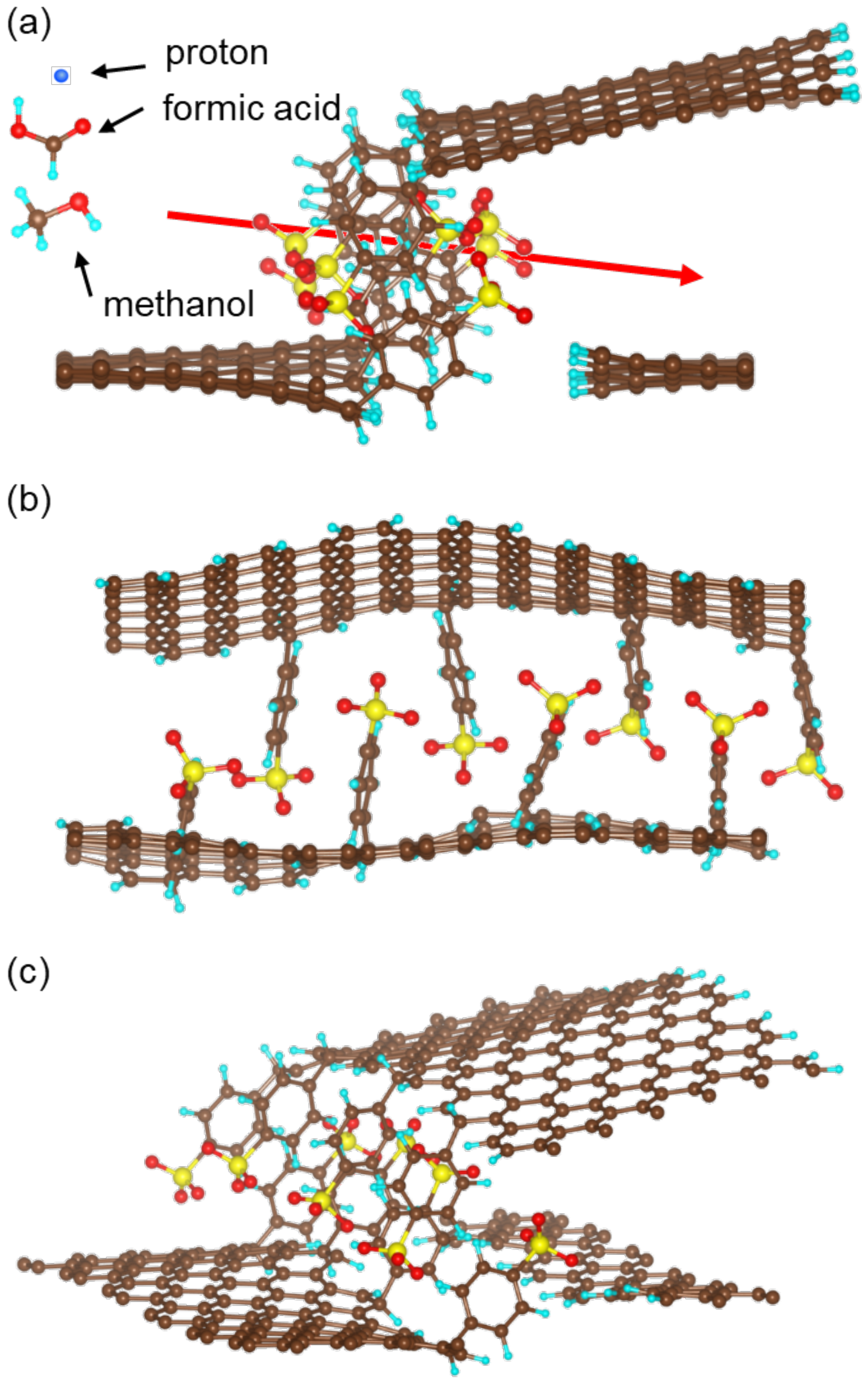

Figure. S23. DFT models of sulfanilic functionalized groups on the graphene edge. Each model (a-c) presents different view angles.

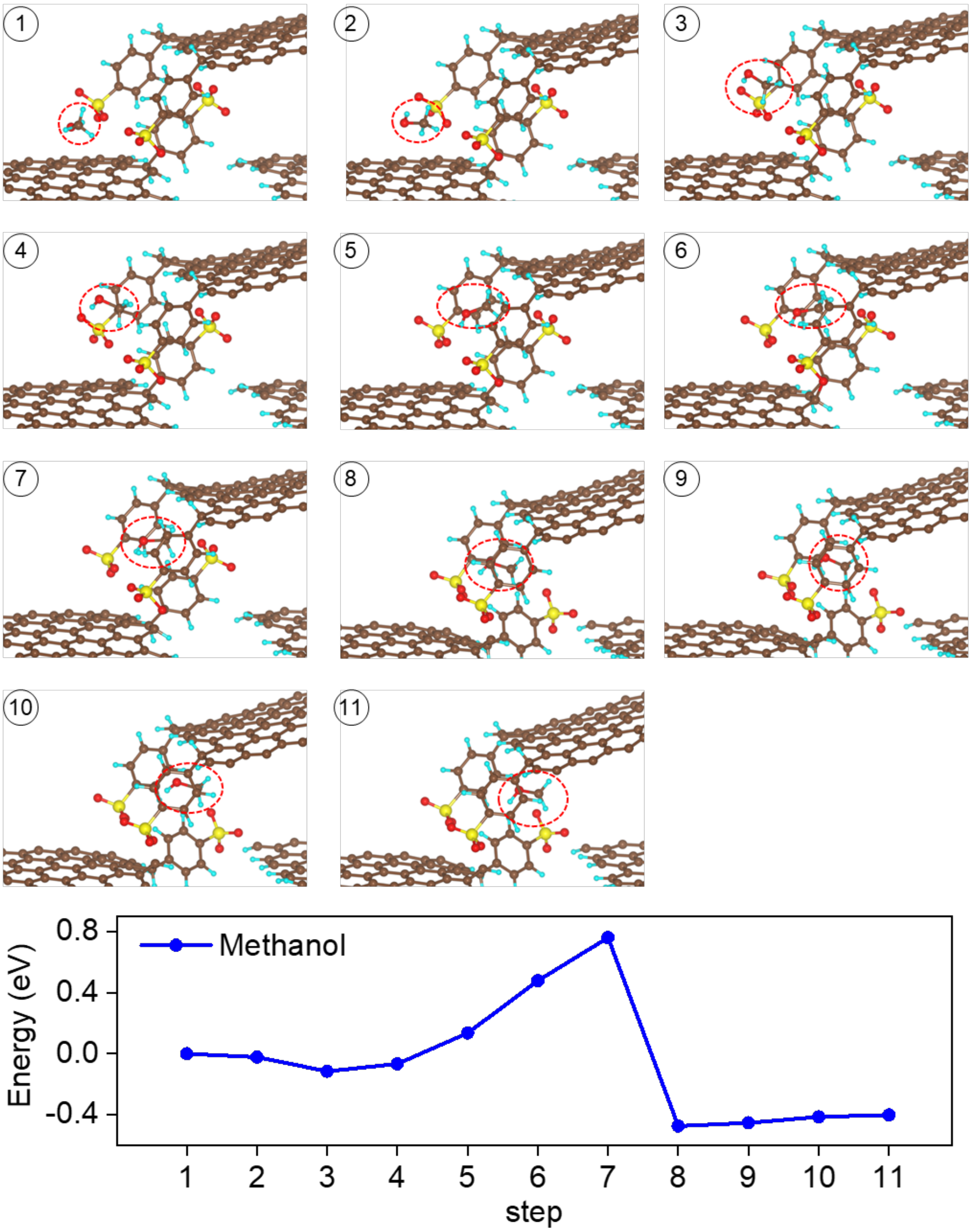

Figure. S24. DFT models of methanol passage through sulfanilic functionalized groups on the graphene edge. The graph plots present their energies for each step.

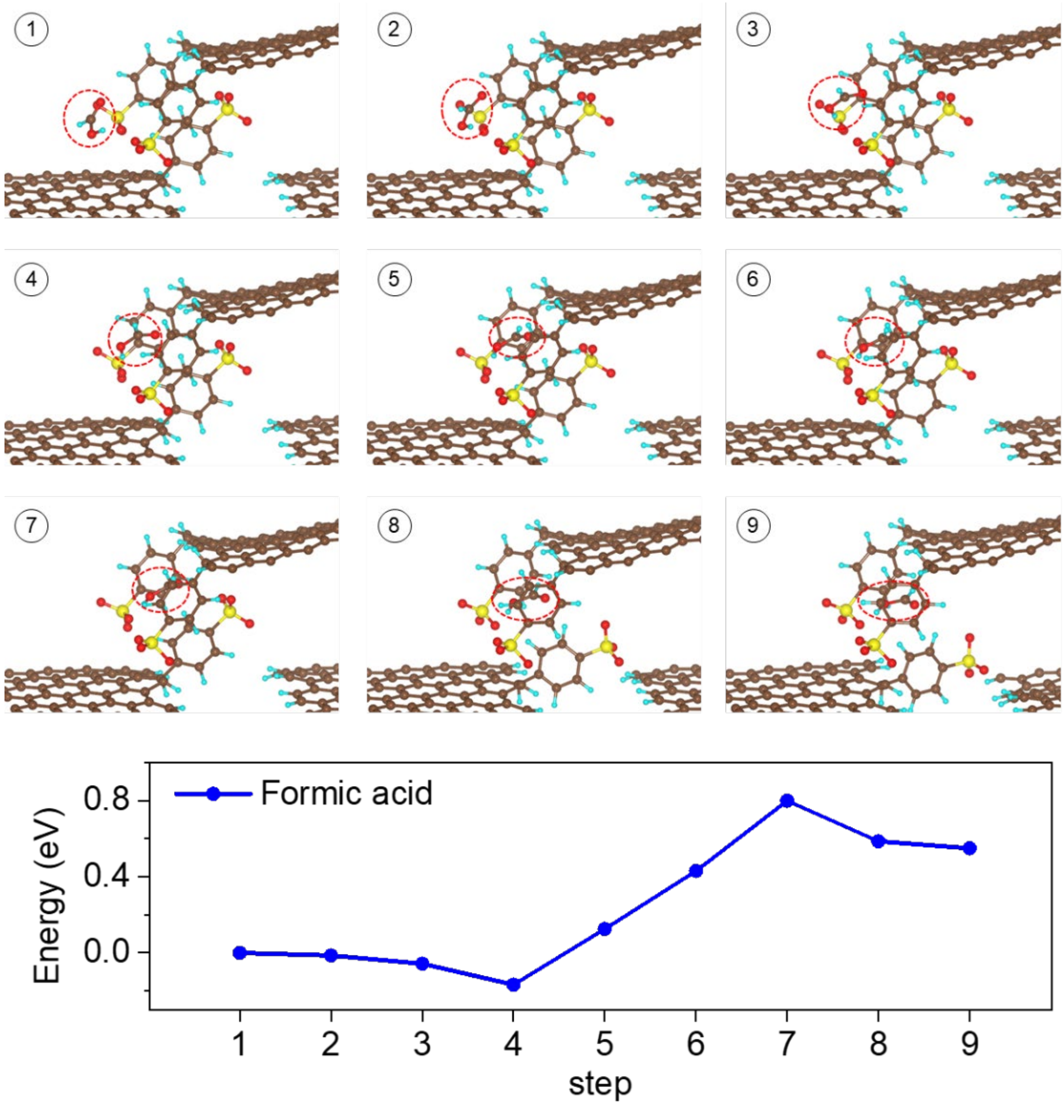

Figure. S25. DFT models of formic acid passage models through sulfanilic functionalized groups on the graphene edge. The graph plots present their energies for each step.

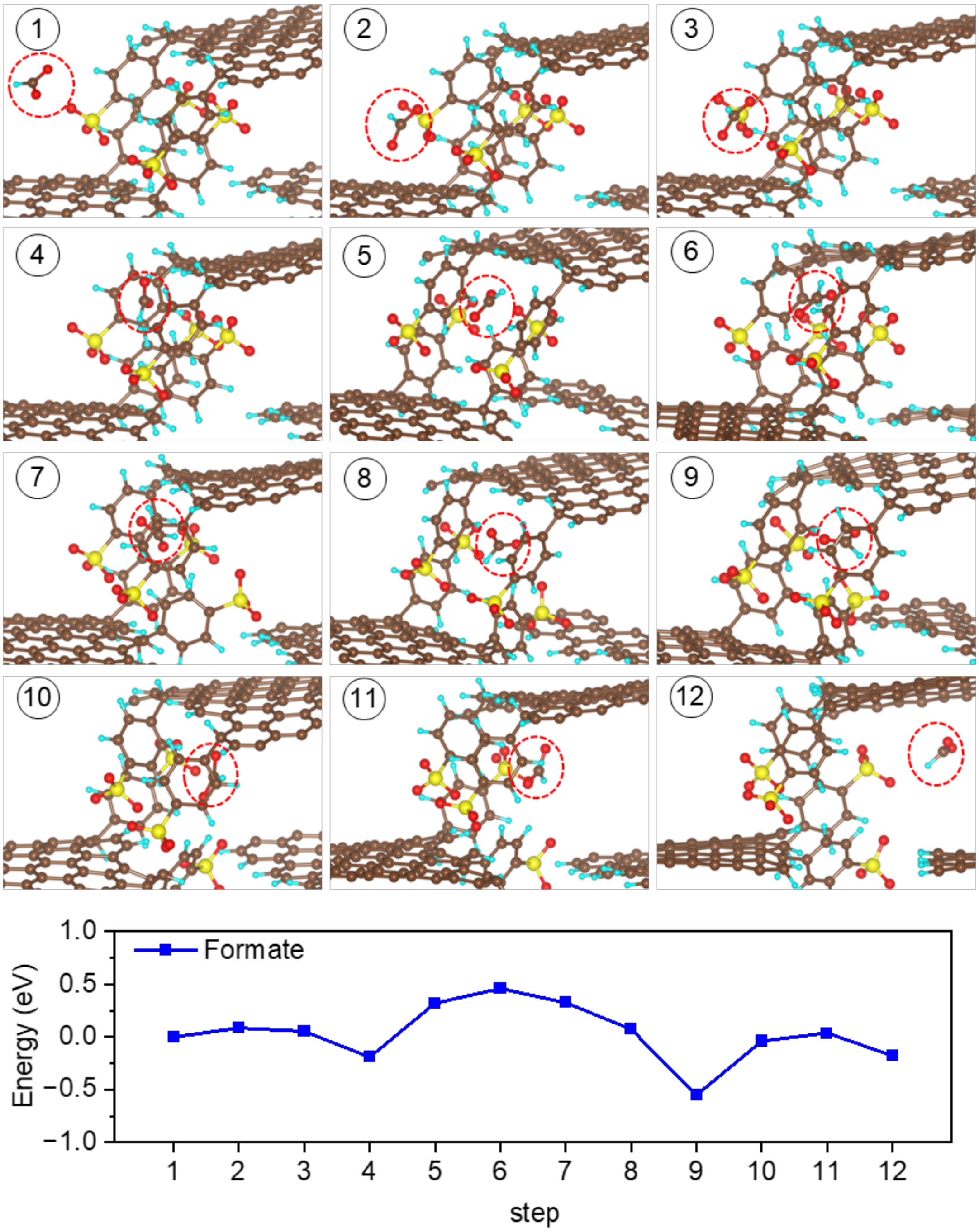

Figure. S26. DFT models of formate passage models through sulfanilic functionalized groups on the graphene edge. The graph plots present their energies for each step.

Table. S1. Summary of areal proton conductivity and methanol and formate crossover rates for GLs, hGLs, fGLs, spin-coated Nafion, Nafion®117, Nafion®212, and bare hole without any membranes.

	**Areal proton conductivity (mS cm^{-2})**	**Crossover rate (mol m^{-2} s^{-1})**	
		Methanol	Formate
**N/1GL/N**	0.135	0.530	0.098
**N/2GL/N**	0.034	0.143	0.065
**N/3GL/N**	0.016	0.095	0.039
**N/1hGL/N**	1060	0.715	0.163
**N/2hGL/N**	801	0.290	0.130
**N/3hGL/N**	509	0.218	0.097
**N/1fGL/N**	1500	0.573	0.163
**N/2fGL/N**	1232	0.169	0.117
**N/3fGL/N**	818	0.120	0.052
**Nafion/Nafion (N/N)**	1930	1.060	0.261
**Bare hole without any membranes**	3900	1.700	1.12
**N/3fGL/Nafion®117**	53.9	0.0053	0.0007
**Nafion®117**	88.7	0.0184	0.0015
**N/3fGL/Nafion®212**	80.7	0.0177	0.0060
**Nafion®212**	117.2	0.0425	0.0146

Table. S2. Summary of areal proton conductivities and crossover suppression rate of methanol and formate on various membranes. The crossover suppression ratio of graphene-pasted Nafion to Nafion without graphenes indicates how much the methanol/formic acid crossover rate was suppressed by the Nafion ratio.

	**Areal proton conductivity (mS/cm^2)**	**Crossover suppression ratio**	
		**Methanol (%)**	**Formate (%)**
**N/3fGL/Nafion®117*a**	53.9	71.5	53.2
**N/3fGL/Nafion®212*b**	80.7	58.5	58.7
**N/1GL/N**	0.135	50.0	62.5
**N/2GL/N**	0.034	86.5	75.0
**N/3GL/N**	0.016	91.0	85.0
**N/1hGL/N**	1060	32.5	37.5
**N/2hGL/N**	801	72.6	50.2
**N/3hGL/N**	509	79.4	62.8
**N/1fGL/N**	1500	45.9	37.5
**N/2fGL/N**	1232	84.1	55.2
**N/3fGL/N**	818	88.7	80.0

*a: compared with Nafion®117, *b: compared with Nafion®212, and other membrane were compared with our spin-coated Nafion.

Table. S3. Summary of calculated membrane performance values for proton conductivity and methanol based on equation S1.

Sample	Membrane performance (MP)	Sample	Membrane performance (MP)
**N/1GL/N*a**	−3.85	**Graphene/hBN[7]**	−0.14
**N/2GL/N*a**	−3.88	**AMA nanosheet[8]**	0.23
**N/3GL/N*a**	−4.03	**PFSA membrane[9]**	0.54
**N/1hGL/N*a**	−0.09	**Nafion-fullerene[10]**	0.28
**N/2hGL/N*a**	0.18	**Nafion-hBN[11]**	0.37
**N/3hGL/N*a**	0.11	**SPSU/FPGO[12]**	0.22
**N/1fGL/N*a**	0.16	**SPEEK[13]**	−0.35
**N/2fGL/N*a**	0.60	**c-SPI[14]**	0.64
**N/3fGL/N*a**	0.57	**CS-SiO_2[15]**	0.14
**Nafion®117*a**	0.42	**SPI[16]**	0.02
**N/3fGL/Nafion®117*a**	0.75	**Nafion (reference)**	0.0
**N/3fGL/Nafion®117*b**	0.33		
**Nafion®212*a**	0.18		
**N/3fGL/Nafion®212*a**	0.40		
**N/3fGL/Nafion®212*c**	0.22		

*a: normalized by our Nafion/Nafion, *b: normalized by Nafion®117, *c: normalized by Nafion®212, and other membranes were normalized by each Nafion measured on each report.

Table. S4. Summary of calculated membrane performance values for proton conductivity and formate based on equation S1.

Sample	Membrane performance (MP)
**N/1GL/N*a**	−3.73
**N/2GL/N*a**	−4.15
**N/3GL/N*a**	−4.26
**N/1hGL/N*a**	−0.06
**N/2hGL/N*a**	−0.08
**N/3hGL/N*a**	−0.15
**N/1fGL/N*a**	0.09
**N/2fGL/N*a**	0.15
**N/3fGL/N*a**	0.33
**Nafion®117*a**	0.89
**N/3fGL/Nafion®117*a**	1.01
**N/3fGL/Nafion®117*b**	0.11
**Nafion®212*a**	0.03
**N/3fGL/Nafion®212*a**	0.27
**N/3fGL/Nafion®212*c**	0.22
**Nafion (reference)**	0.00

*a: normalized by our Nafion/Nafion, *b: normalized by Nafion®117, *c: normalized by Nafion®212, and other membranes were normalized by each Nafion measured on each report.

Table. S5. Experimental values of energy barrier of proton, methanol, and formate penetration through N/3hGL/N and N/3fGL/N.

	**Proton penetration at −1.0 V**	**Methanol penetration at −1.6 V**	**Formate penetration at −1.6 V**
**N/3hGL/N**	0.37 eV	0.30 eV	0.19 eV
**N/3fGL/N**	0.13 eV	0.36 eV	0.34 eV

Table. S6. DFT-calculated values of the energy barrier for proton and formate penetration through nano-hole graphene sheet.

**Hole size**	**φ 0.36 nm**	**φ 0.8 nm**
**Proton**	0.15 eV	0 eV
**Formic acid**	5.1 eV	−0.4 eV

Table. S7. DFT-calculated values of the energy barrier for the proton, formic acid, and methanol passage through interlayer graphene.

	**Energy barrier**
**Proton**	−0.63 eV
**Methanol**	0.36 eV
**Formic acid**	0.82 eV

Table. S8. DFT-calculated values of the energy barrier for the proton, methanol, and formic acid passage through sulfanilic functionalized groups on the graphene edge.

	**Energy barrier**
**Proton**	0.21 eV
**Methanol**	0.76 eV
**Formic acid**	0.80 eV

References

[1] K. L. Hu, T. Ohto, Y. Nagata, M. Wakisaka, Y. Aoki, J. Fujita, Y. Ito, *Nat. Commun* **2021**, *12* (1), 9, https://doi.org/10.1038/s41467-020-20503-7.

[2] C. H. Lee, M. W. Kanan, *ACS Catal.* **2015**, *5* (1), 465, https://doi.org/10.1021/cs5017672; W. C. Ma, S. J. Xie, X. G. Zhang, F. F. Sun, J. C. Kang, Z. Jiang, Q. H. Zhang, D. Y. Wu, Y. Wang, *Na. Commun* **2019**, *10*, 10, https://doi.org/10.1038/s41467-019-08805-x; Y. H. He, W. J. Jiang, Y. Zhang, L. B. Huang, J. S. Hu, *J. Mater. Chem. A* **2019**, *7* (31), 18428, https://doi.org/10.1039/c9ta05937g; J. J. Wu, F. G. Risalvato, F. S. Ke, P. J. Pellechia, X. D. Zhou, *J. Electrochem. Soc.* **2012**, *159* (7), F353, https://doi.org/10.1149/2.049207jes.

[3] K. P. Kuhl, E. R. Cave, D. N. Abram, T. F. Jaramillo, *Energy Environ. Sci.* **2012**, *5* (5), 7050, https://doi.org/10.1039/c2ee21234j.

[4] S. Jeong, T. Ohto, T. Nishiuchi, Y. Nagata, J. Fujita, Y. Ito, *ACS Catal.* **2021**, *11* (15), 9962, https://doi.org/10.1021/acscatal.1c02646.

[5] T. Tsujiguchi, Y. Kawabe, S. Jeong, T. Ohto, S. Kukunuri, H. Kuramochi, Y. Takahashi, T. Nishiuchi, H. Masuda, M. Wakisaka, K. L. Hu, G. Elumalai, J. I. Fujita, Y. Ito, *ACS Catal.* **2021**, *11* (6), 3310, https://doi.org/10.1021/acscatal.0c04887.

[6] J. B. Wu, M. L. Lin, X. Cong, H. N. Liu, P. H. Tan, *Chem. Soc. Rev.***2018**, *47* (5), 1822, https://doi.org/10.1039/c6cs00915h; Z. Z. Miao, X. L. Li, L. J. Zhi, *RSC Adv* **2016**, *6* (63), 58561, https://doi.org/10.1039/c6ra12470d.

[7] S. M. Holmes, P. Balakrishnan, V. S. Kalangi, X. Zhang, M. Lozada-Hidalgo, P. M. Ajayan, R. R. Nair, *Adv. Energy. Mat.* **2017**, *7* (5), 7, https://doi.org/10.1002/aenm.201601216.

[8] X. L. Guo, Y. Fan, J. N. Xu, L. Wang, J. F. Zheng, *Ind. Eng. Chem. Res.* **2020**, *59* (33), 14825, https://doi.org/10.1021/acs.iecr.0c02741.

[9] A. S. Arico, D. Sebastian, M. Schuster, B. Bauer, C. D'Urso, F. Lufrano, V. Baglio, *Membranes* **2015**, *5* (4), 793, https://doi.org/10.3390/membranes5040793.

[10] G. Rambabu, N. Nagaraju, S. D. Bhat, *Chem. Eng. J.* **2016**, *306*, 43, https://doi.org/10.1016/j.cej.2016.07.032.

[11] V. Parthiban, A. K. Sahu, *New J. Chem.* **2020**, *44* (18), 7338, https://doi.org/10.1039/d0nj00433b.

[12] T. J. Yang, Z. L. Li, H. L. Lyu, J. J. Zheng, J. L. Liu, F. N. Liu, Z. Y. Zhang, H. X. Rao, *RSC Adv* **2018**, *8* (28), 15740, https://doi.org/10.1039/c8ra01731j.

[13] L. Li, J. Zhang, Y. X. Wang, *J. Mater. Sci. Lett.* **2003**, *22* (22), 1595, https://doi.org/10.1023/a:1026336524991.

[14] E. Higuchi, N. Asano, K. Miyatake, H. Uchida, M. Watanabe, *Electrochim. Acta* **2007**, *52* (16), 5272, https://doi.org/10.1016/j.electacta.2007.02.056.

[15] J. H. Kim, S. K. Kim, K. Nam, D. W. Kim, *J. Membr. Sci.* **2012**, *415*, 696, https://doi.org/10.1016/j.memsci.2012.05.057.
[16] Y. Yin, J. H. Fang, Y. F. Cui, K. Tanaka, H. Kita, K. Okamoto, *Polymer* **2003**, *44* (16), 4509, https://doi.org/10.1016/s0032-3861(03)00439-7.
